# High‐throughput sequencing on preservative ethanol is effective at jointly examining infraspecific and taxonomic diversity, although bioinformatics pipelines do not perform equally

**DOI:** 10.1002/ece3.7453

**Published:** 2021-03-23

**Authors:** Marjorie Couton, Aurélien Baud, Claire Daguin‐Thiébaut, Erwan Corre, Thierry Comtet, Frédérique Viard

**Affiliations:** ^1^ Sorbonne université CNRS UMR 7144 Station Biologique de Roscoff Roscoff France; ^2^ Sorbonne université CNRS FR 2424 Station Biologique de Roscoff Roscoff France; ^3^ ISEM Univ Montpellier CNRS EPHE IRD Montpellier France

**Keywords:** biodiversity, bioinformatics, bulkDNA, ethanol‐based DNA, haplotype diversity, high‐throughput sequencing, metabarcoding, tunicate

## Abstract

1. High‐throughput sequencing of amplicons (HTSA) has been proposed as an effective approach to evaluate taxonomic and genetic diversity at the same time. However, there are still uncertainties as to how the results produced by different bioinformatics treatments impact the conclusions drawn on biodiversity and population genetics indices.
2. We evaluated the ability of six bioinformatics pipelines to recover taxonomic and genetic diversity from HTSA data obtained from controlled assemblages. To that end, 20 assemblages were produced using 354 colonies of *Botrylloides* spp., sampled in the wild in ten marinas around Brittany (France). We used DNA extracted from preservative ethanol (ebDNA) after various time of storage (3, 6, and 12 months), and from a bulk of preserved specimens (bulkDNA). DNA was amplified with primers designed for targeting this ascidian genus. Results obtained from HTSA data were compared with Sanger sequencing on individual zooids (i.e., individual barcoding).
3. Species identification and relative abundance determined with HTSA data from either ebDNA or bulkDNA were similar to those obtained with traditional individual barcoding. However, after 12 months of storage, the correlation between HTSA and individual‐based data was lower than after shorter durations. The six bioinformatics pipelines were able to depict accurately the genetic diversity using standard population genetics indices (H_S_ and F_ST_), despite producing false positives and missing rare haplotypes. However, they did not perform equally and dada2 was the only pipeline able to retrieve all expected haplotypes.
4. This study showed that ebDNA is a nondestructive alternative for both species identification and haplotype recovery, providing storage does not last more than 6 months before DNA extraction. Choosing the bioinformatics pipeline is a matter of compromise, aiming to retrieve all true haplotypes while avoiding false positives. We here recommend to process HTSA data using dada2, including a chimera‐removal step. Even if the possibility to use multiplexed primer sets deserves further investigation to expand the taxonomic coverage in future similar studies, we showed that primers targeting a particular genus allowed to reliably analyze this genus within a complex community.

## Graphical abstract

High‐throughput sequencing was applied to DNA extracted from ethanol used to preserve a mixture of field‐collected specimens of the tunicate genus *Botrylloides*. We compared six bioinformatics pipelines to retrieve jointly taxonomic and haplotypic data. All correctly described the species distribution, as compared to standard barcoding. Although they differ in the number of false positives and negatives retrieved, all depicted accurately the genetic diversity using standard population genetics indices.

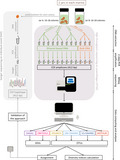

## 1 INTRODUCTION

Although most biodiversity assessments rely on taxonomic diversity, many other components (functional, phylogenetic, genetic…), potentially uncorrelated, are crucial for an exhaustive biodiversity assessment (Lindegren et al., 2018). In this context, high‐throughput sequencing (HTS) of mixed DNAs (Makiola et al., 2020) could be an interesting tool as it offers the possibility to analyze simultaneously two biodiversity components (i.e., taxonomic and genetic). The advantages of this approach also include solving problems related to morphology‐based identification and decreasing handling time and costs as compared to individual‐based methods.

The HTS of amplicons has been tested for studying both taxonomic and genetic diversity, either with primers targeting a broad taxonomic coverage (Elbrecht et al., 2018; Pedro et al., 2017; Stat et al., 2017), or by focusing on one or a few species using primers amplifying a narrower taxonomic range (Marshall & Stepien, 2019; Parsons et al., 2018; Sigsgaard et al., 2017; Stepien et al., 2019; Tsuji, Maruyama, et al., 2020; Tsuji, Miya, et al., 2020). In metazoans, the COI mitochondrial gene has been preferentially used for such studies (e.g., Pedro et al., 2017), because of its high taxonomic resolution, its ability to reveal within‐species polymorphism (Andújar et al., 2018; Bucklin et al., 2011), and because a considerable amount of sequences are available in public databases (Porter & Hajibabaei, 2018). Overall, HTS studies revealed that the most abundant haplotypes (i.e., unique sequences) are easily recovered, some rare ones can be missed, and some spurious sequences can be misidentified as haplotypes. Previous reports showed that different bioinformatics pipelines may produce divergent results regarding taxonomic diversity, especially for species richness (Calderón‐Sanou et al., 2020; Pauvert et al., 2019), but to our knowledge, the consequences of the choice of different algorithms (e.g., clustering *versus* denoising) on haplotype recovery, as well as the impact of the resulting false positives and negatives on commonly used population genetics indices, have not been investigated.

Biodiversity assessments using HTS usually involve the homogenization of all organisms sampled from the target community to extract DNA from bulk. Processing each sample can be time‐consuming and increases the risk of cross‐contamination. Furthermore, this technique implies the destruction of the samples. Shokralla et al. (2010) first showed that preservative ethanol could be used to recover and sequence invertebrate DNA without impacting the integrity of the samples. DNA extracted from preservative ethanol (ethanol‐based DNA, ebDNA) was used for HTS‐based community analyses in terrestrial (Linard et al., 2016; Marquina et al., 2019; Zenker et al., 2020) and freshwater organisms (Erdozain et al., 2019; Hajibabaei et al., 2012; Martins et al., 2019; Zizka et al., 2019). DNA was extracted after various storage durations (from 12 hr to 15 months) and temperatures (from −25°C to ambient). Although Martins et al. (2019) showed that the yield and quality of ebDNA increased in the first 5–10 days of storage, to our knowledge, no experiment has investigated if HTS could be applied after several months of storage for marine organisms.

In this study, we investigated the two knowledge gaps highlighted above. Our goal was to recommend a methodology for jointly assessing taxonomic and genetic diversity *via* HTS on ebDNA. To this end, we evaluated the effectiveness of six metabarcoding analysis pipelines, based on either clustering or denoising approaches, to recover COI haplotypes and assess population genetic diversity indices. DNA was extracted from preservative ethanol of marine organisms stored at room temperature after up to 12 months. As a case study, we examined biofouling communities from marinas which are composed of many nonindigenous species, a major driver of biodiversity loss.

## 2 MATERIALS AND METHODS

### 2.1 Case study and sampling

**FIGURE 1:**
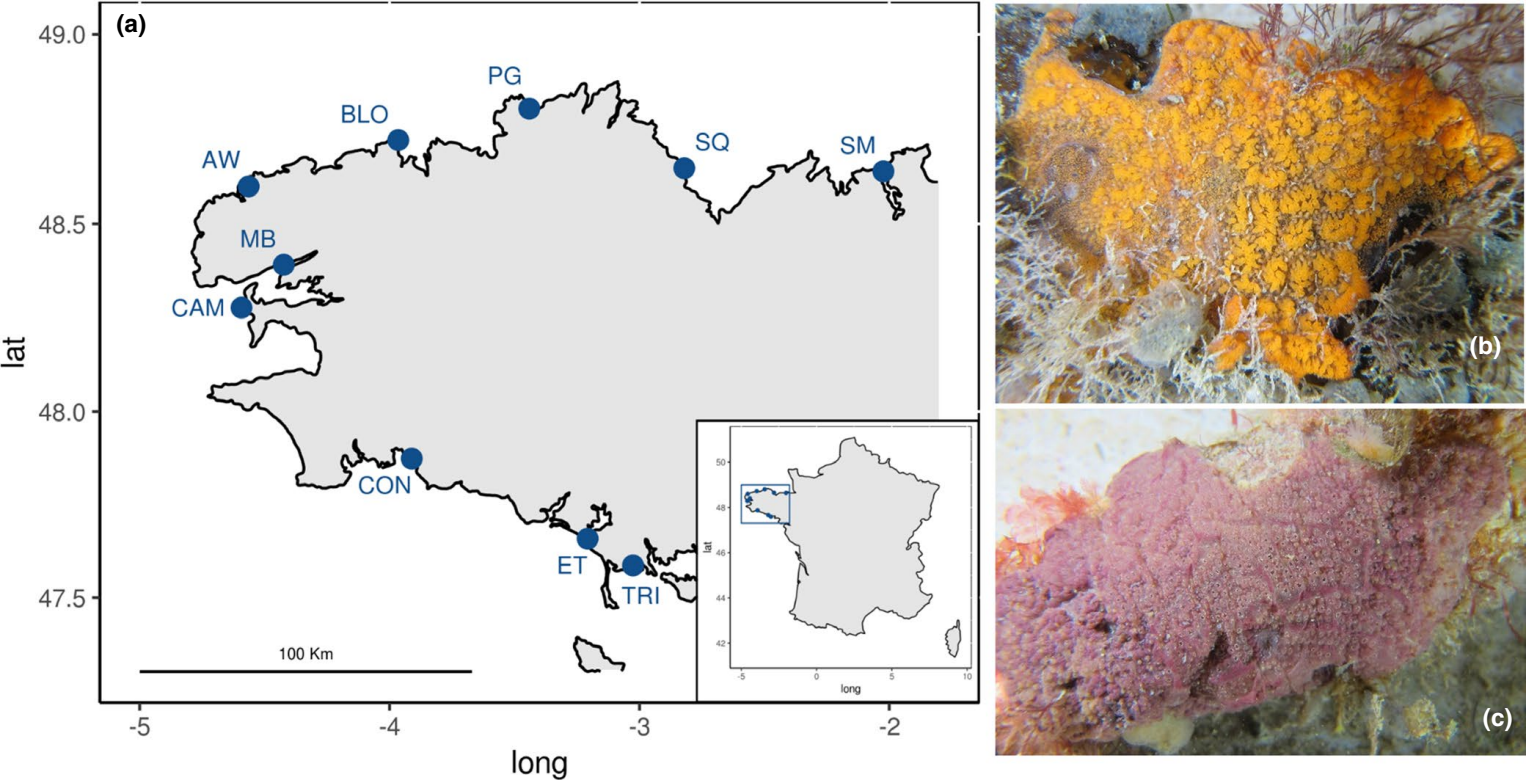
(a) Collection sites of *Botrylloides* spp. SM = Saint‐Malo, SQ = Saint‐Quay‐Portrieux, PG = Perros‐Guirec, BLO = Bloscon (Roscoff), AW = L’Aber‐Wrac'h, MB = Moulin Blanc (Brest), CAM = Camaret‐sur‐Mer, CON = Concarneau, ET = Étel, and TRI = La Trinité‐sur‐Mer. (b) *Botrylloides diegensis*. (c) *Botrylloides violaceus*. Photo credit: Yann Fontana

We selected species of the genus *Botrylloides* as a case study. They are colonial ascidians composed of hundreds of individuals (zooids) embedded in a tunic (Figure 1b,c). Among the 19 accepted species, two from our study area (English Channel), *Botrylloides violaceus* Oka, 1927, and *Botrylloides diegensis* Ritter & Forsyth, 1917, are recognized as globally invasive (Bock et al., 2011; Viard et al., 2019). The native *B. leachii* (Savigny, 1816) has also been reported in our study area, in addition to a cryptic lineage, morphologically undistinguishable from *B. violaceus* (BvX‐H6 after Viard et al., 2019). *Botrylloides* species are notoriously difficult to identify based on morphology (Rocha et al., 2019; Viard et al., 2019). The COI marker is, however, effective in discriminating species from this genus (Rocha et al., 2019) and in detecting infraspecific diversity for these taxa (Viard et al., 2019).

*Botrylloides* colonies were sampled by scuba diving in 10 marinas around Brittany (English Channel and NE Atlantic, France; Figure 1a). Between 32 and 36 colonies were collected haphazardly in each location along a 100‐m transect below pontoons. A small piece of each colony was isolated in 100% ethanol for individual haplotype identification. The remaining parts of the colonies were stored together in 2‐L plastic jars filled with 100% ethanol for further HTS‐based analyses, at room temperature. To limit potential biases that might arise from different biomass, larger colonies were resized, before preservation, to roughly similar sizes. The ethanol/tissue ratio was optimized by distributing the colonies into two jars (A and B) per marina.

### 2.2 Sanger sequencing on individual zooid (SSIZ)

For each piece of colony preserved individually, DNA was extracted from a single zooid using the NucleoSpin® Tissue extraction kit (Macherey‐Nagel) (Appendix S1 [SI.1]). A 709‐bp (with primers) portion of the COI gene was amplified using primers designed by Folmer et al. (1994). Because these primers are not always effective in amplifying *Botrylloides* species, all individuals with a poor sequencing quality (59 *B. diegensis* and 17 *B. violaceus*) were additionally amplified with primers targeting each species. The first pair was designed by Callahan et al. (2010) for *B. violaceus* (644 bp), and the second pair was newly designed [Bdieg‐COI‐F: 5′‐TGTCTACTAATCATAAAGATATTAG‐3′; Bdieg‐COI‐R2: 5′‐AATATACACTTCAGGGTGTCCAA‐3′] for *B. diegensis* (713 bp). Both target the same COI region as Folmer et al.’s primers. Details are provided in Appendix S1 (SI.1). Amplicons were sequenced in both directions by Eurofins Genomics (Germany GmbH) using Sanger technology. Sequences were aligned using CodonCode Aligner v.5.0.1 (CodonCode Corporation, Dedham, MA). Species identification and haplotype names were provided according to Viard et al. (2019), using consecutive numbers for newly discovered haplotypes.

### 2.3 High‐throughput sequencing on assemblages (HTSA)

#### 2.3.1 Sample processing

**FIGURE 2:**
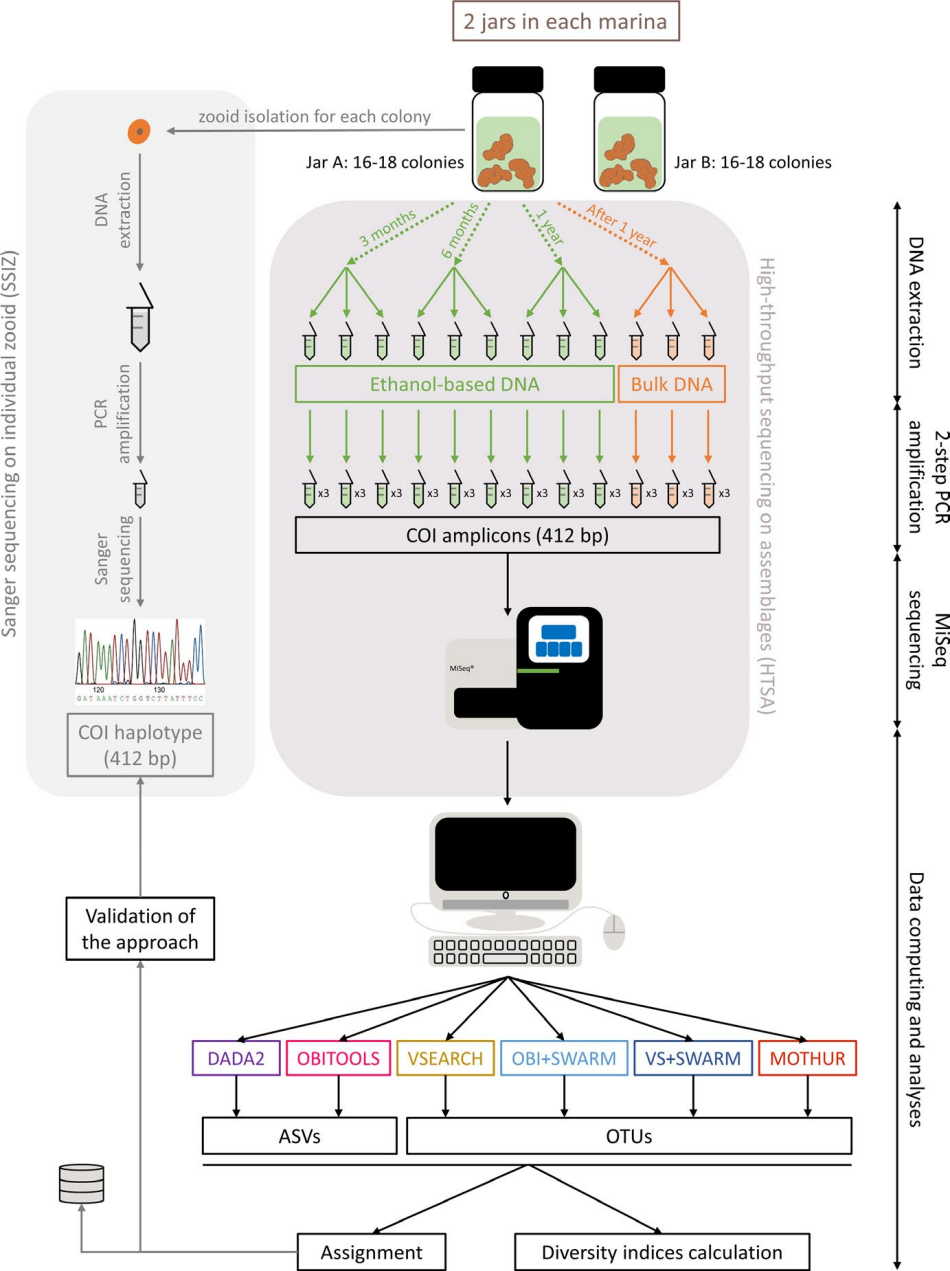
Overview of the experimental design from DNA extraction to data analyses. Dotted arrows represent the four types of samples (3‐, 6‐, and 12‐month ebDNA and bulkDNA). Data were processed with six bioinformatics pipelines. Extraction and amplification protocols are detailed as Appendix S1

After 3, 6, and 12 months of storage, DNA was extracted from preservative ethanol (ebDNA), with three replicates of 1 ml per jar (Figure 2). In addition, after 12 months, all colonies from a jar were blended, and DNA was extracted (bulkDNA) in three replicates (Figure 2; Appendix S1 [SI.2]).

Because primers targeting a broad taxonomic range are commonly prone to amplification biases, novel primers were designed to ensure the amplification of *Botrylloides* species. The fragment obtained with SSIZ being too long for Illumina sequencing, primers were designed to target a shorter 455‐bp portion, sufficient to recover all known haplotypes (Viard et al., 2019; this study): COIBotrF2.2 ‐ 5′‐AGTGTTTTYATTCGTWTAGA‐3′ and COIBotrR7.1 ‐ 5′‐CAAAACARAGAYATRGARAAYAT‐3′. The reliability of this primer pair was tested by PCR amplification of template DNA from several ascidian species and by in silico PCR using ecoPCR from the obitools‐1.2.11 (Boyer et al., 2016; Appendix S1 [SI.3]). The libraries were prepared using a dual‐barcoded, dual‐indexed two‐step PCR procedure (Bourlat et al., 2016) detailed in Appendix S1 (SI.4). Briefly, each extraction replicate was amplified using three tagged primer combinations. Three PCR products amplified with the same tagged primer combination were pooled. This resulted in a total of nine technical tagged replicates (i.e., three tagged PCR replicates for each of the three extraction replicates) per sample. Then, all tagged PCR products for a given type of sample (Figure 2) were pooled and a second PCR was performed to add Nextera® indexed primers. Each sample was identified by a unique index combination. All amplicons were sequenced in‐house using a MiSeq® Illumina instrument with a v3 Reagent Kit (600 cycles).

#### 2.3.2 Reads processing

The HTSA dataset was processed using six pipelines (Figure 2), based either on denoising algorithms, which remove PCR and sequencing errors and produce amplicon sequence variants (ASVs), or on clustering algorithms producing operational taxonomic units (OTUs). These two approaches have their own benefits for analyzing infraspecific diversity; clustering is expected to be less effective than denoising in identifying haplotypes, thus producing more false negatives, but should retain less false positives. The denoising‐based pipelines were dada2 v‐1.13.1 (Callahan et al., 2016) and obitools v‐1.2.11. The four others were clustering‐based. vsearch v‐2.14.1 (Rognes et al., 2016) and mothur v‐1.42.0 (Schloss et al., 2009) require an arbitrary identity threshold for clustering, whereas swarm v‐3.0.0 is based on multiple local identity thresholds (Mahé et al., 2015). Since swarm only offers a clustering tool, reads preparation was performed with either the obitools (obi + swarm) or the vsearch (vs + swarm) processing tools. Parameter choice is of critical importance to get optimal results. As for a regular study, where the sample composition is not known a priori, the parameter values were first selected to be the most sensitive and effective in retrieving highly similar haplotypes. For further evaluation of the impact of these parameters on the amount of false positives and false negatives, other sets of values were tested. The overall conclusions remained unchanged with these other settings (Appendix S1 [SI.5]), so the detailed results shown in the next sections were those obtained with the first parameter settings.

False positives may arise from index‐jump (Taberlet et al., 2018). To assess this phenomenon, 12 index combinations not used in our PCR experiments were added to the MiSeq sequencing sample sheet in order to get the corresponding fastq files. The number of reads associated to these internal control index combinations was recorded (maximum 25–37 reads depending on the pipeline). Any ASV or OTU that did not account for more than twice the maximum number of reads in a control index combination was discarded. Furthermore, we retained only ASVs/OTUs found in at least five out of the nine technical replicates per sample.

### 2.4 Data analyses

#### 2.4.1 Assignment

ASVs/OTUs were compared to a database composed of 1,107 reference sequences for 185 tunicate species collected from GenBank or produced locally (Couton et al., 2019). It included all known haplotypes from the three local *Botrylloides* species and BvX‐H6 (Viard et al., 2019), as well as two new haplotypes found with SSIZ. Species assignment was performed using the Blast® command‐line tool (Altschul et al., 1990). Only alignments covering 99% of the subject sequence were considered. If one ASV/OTU matched with several references, it was assigned to the one with the highest identity percentage. If two alignments with different references had the same identity, the ASV/OTU was classified as “unassigned.” For assignment at the haplotype level, only ASVs/OTUs which were 100% identical to one of the known haplotypes were assigned.

#### 2.4.2 Haplotype comparison

The proportion of reads assigned to a given haplotype in a jar was compared to the proportion of colonies associated to this haplotype by SSIZ in the same jar, using Pearson correlation with R‐3.4.4 (R Core Team, 2018). The effect of the pipeline and type of sample on the correlation coefficient (*r*) was tested by a Friedman test with R‐3.4.4. For each factor, pairwise comparisons were done with a paired Wilcoxon test, with *p*‐values adjusted for multiple comparisons. For picturing the molecular distance between known and unassigned ASVs/OTUs, haplotype networks were built with the pegas v‐0.10 R package (Paradis, 2010).

#### 2.4.3 Diversity indices

To evaluate the reliability of ASV/OTU frequencies as infraspecific diversity descriptors, two common population genetics indices were estimated: (a) the average gene diversity per locus (H_S_; Nei, 1973) and (b) the population pairwise F_ST_ estimator (Weir & Cockerham, 1984), a measure of the genetic structure. Only ASVs/OTUs assigned to *B. diegensis,* the most conspicuous species, were used. Computations were made using arlequin 3.5.2.2 (Excoffier & Lischer, 2010) with either the haplotype frequencies from SSIZ or the ASV/OTU frequencies, per marina (jars pooled), from each pipeline. Pearson correlation coefficients between H_S_ from SSIZ and HTSA datasets were computed. The effect of the pipeline or the type of sample on correlation coefficients was tested by a Friedman test, and for each factor, pairwise comparisons were done with a paired Wilcoxon test, with *p*‐values adjusted for multiple comparisons. Pairwise F_ST_ matrices obtained with HTSA and SSIZ were compared using a Mantel test with the vegan‐2.5.2 R package (Oksanen et al., 2018), and Pearson correlations were computed. Pairwise F_ST_ estimators from SSIZ and HTSA, on 3‐month ebDNA processed with dada2, were used to build a heatmap with ggplot2‐3.1.1 (Wickham, 2016) and dendrograms with the hclust function (method UPGMA) in R‐3.4.4 and ggdendro‐0.1‐20 (De Vries & Ripley, 2016).

## 3 RESULTS

### 3.1 Sanger sequencing on individual zooid (SSIZ)

**FIGURE 3:**
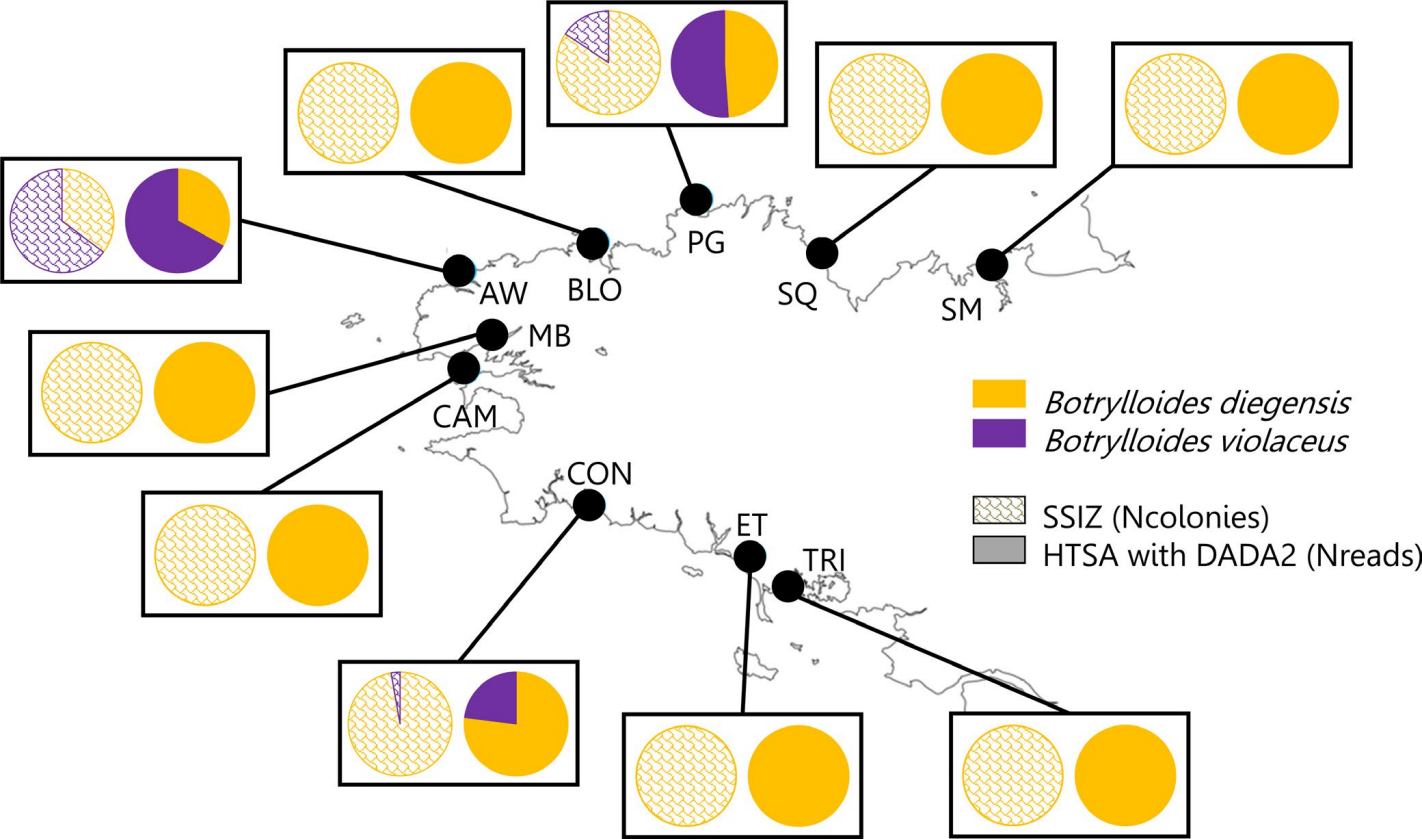
Distribution patterns of *Botrylloides diegensis* (yellow) and *Botrylloides violaceus* (purple) as uncovered by SSIZ (scale pattern) or HTSA (results from dada2 3‐month ebDNA; plain color). See Figure 1 for location codes

Out of the 354 colonies, 353 were successfully amplified. The one that failed was later assigned to *Botrylloides violaceus* with cytochrome b (not shown). Only the two non‐ indigenous species *B. diegensis* and *B. violaceus* were present, *B. diegensis* being the most abundant (92% of the colonies; Figure 3). Across the two species and all samples, nine haplotypes were found. In *B. diegensis*, five (Bd‐H1, Bd‐H2, Bd‐H3, Bd‐H5, and Bd‐H6) were already reported in Viard et al. (2019), and two were new (Bd‐H7 and Bd‐H8). In *B. violaceus*, the two haplotypes (Bv‐H1 and Bv‐H4) were already reported.

### 3.2 Species assignment

None of the four negative controls of extraction and PCR contained any reads after the filtering steps. The MiSeq run yielded 11,695,927 reads that resulted in 61 unique ASVs/OTUs, some being shared across methods. All ASVs/OTUs were assigned to either *B. diegensis* or *B. violaceus* with more than 97% identity, 45 being assigned with more than 99% identity. The 16 ASVs/OTUs with less than 99% identity accounted for only 2% of the total amount of reads. In agreement with SSIZ, HTSA revealed the presence of *B. diegensis* in every location, whereas *B. violaceus* was detected in three marinas only (PG, AW, and CON; Figure 3). The proportions of both species estimated from HTSA and SSIZ significantly differed in PG for ebDNA samples and CON for all types of samples, except 6‐month ebDNA, with four pipelines, but did not differ in AW (Fisher's exact test; Table S5). When different, HTSA always overestimated the abundance of *B. violaceus*.

### 3.3 Pipeline performance for HTSA‐based haplotype detection

**FIGURE 4:**
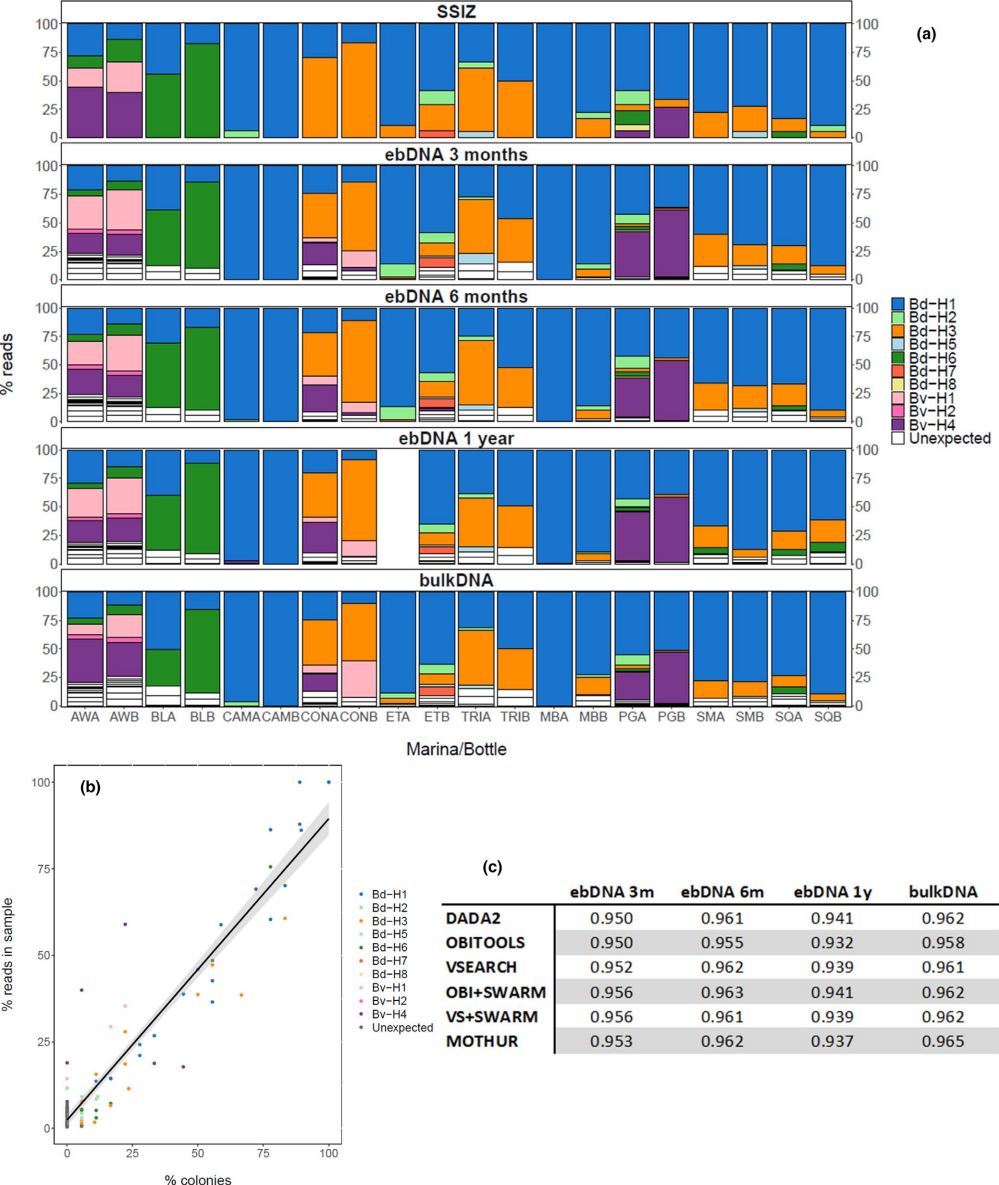
(a) Proportion of colonies or reads per haplotype in each jar (A and B) for each location (codes in Figure 1), as revealed by SSIZ (top panel) or HTSA using dada2 for the four types of samples (four lower panels). One‐year ebDNA for ETA could not be amplified. (b) Correlation between the proportion of reads (dada2, 3‐month ebDNA) and the proportion of colonies (SSIZ) of a given haplotype in the same jar, 95% confidence interval in gray. (c) Pearson correlation coefficient for each pipeline and sample type, as shown in b (*p* < .001 for all values)

The six pipelines generated 20–36 ASVs/OTUs (Table 1). This is two to four times the number of haplotypes expected from SSIZ (nine haplotypes). The five dominant haplotypes in SSIZ (Bd‐H1, Bd‐H3, Bd‐H6, Bv‐H1, and Bv‐H4; Figure 4a) were retrieved by all pipelines. dada2 retrieved all nine haplotypes but produced a high number of unexpected ASVs (20), whereas mothur had the lowest number of unexpected sequences (14) but recovered only six expected haplotypes (Table 1). The chosen parameter values showed the highest sensitivity (i.e., allowed the recovery of the highest number of expected haplotypes) for all pipelines except obitools for which another set of values allowed recovering one additional haplotype (Appendix S1 [SI.5]; Table S4) The proportion of reads associated with unexpected sequences was low (1.5%–9%; Table 1), and most of them were not shared between pipelines (Figure S4).

**TABLE 1:** Number of ASVs/OTUs retained with the six pipelines using parameter values chosen a priori (see Materials and Methods), and after post‐treatment corrections (index‐jump and selection on replicates). After comparison with SSIZ results, the number of expected haplotypes recovered, the names of missing haplotypes and the proportion of reads associated with unexpected sequences are indicated. Results for the other settings tested are provided in Appendix S1 (SI.5)

	ASVs/OTUs	Index‐jump correction	Present in at least five replicates	Expected haplotypes recovered	Missing haplotypes	% reads of unexpected sequences
dada2	2,115	58	29	9	‐	9
obitools	4,062	46	23	5	Bd‐H2 Bd‐H5 Bd‐H7 Bd‐H8	5
vsearch	3,055	64	36	7	Bd‐H2 Bd‐H8	8
obi + swarm	896	46	23	5	Bd‐H2 Bd‐H5 Bd‐H7 Bd‐H8	3
vs + swarm	1,386	46	22	5	Bd‐H2 Bd‐H5 Bd‐H7 Bd‐H8	1.5
mothur	3,270	34	20	6	Bd‐H2 Bd‐H5 Bd‐H8	2

**FIGURE 5:**
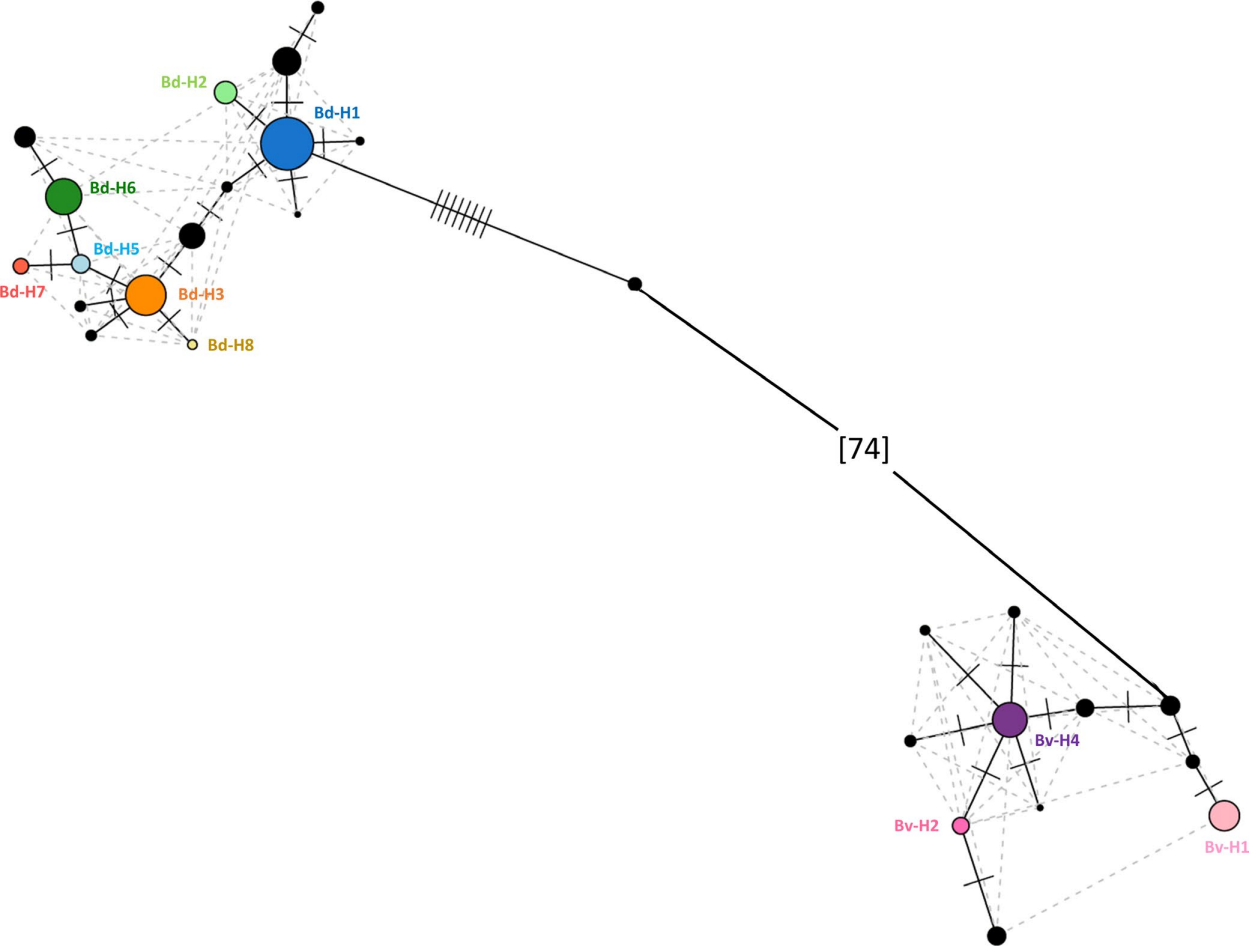
Haplotype network built with ASVs produced by dada2 on 3‐month ebDNA data. Expected haplotypes are in color, and unexpected sequences are in black. The size of the nodes represents the ASV abundance (fourth root of the number of reads) in the dataset. The number of crossing lines represents the number of mutations between two nodes. The dashed gray lines figure alternative links. The 74‐mutation step linking the two species has been shortened for visualization purposes

ASVs obtained with dada2 from 3‐month ebDNA (our recommended pipeline x type of sample combination; see discussion) were used to compute a haplotype network (Figure 5). With one exception, all unexpected sequences differed by only one or two nucleotides from expected haplotypes. The ASV with an 8 bp difference from Bd‐H1 was a chimera: the 381 first bases corresponded to Bd‐H1 and the last 31 bases corresponded to Bv‐H4 or Bv‐H1. This sequence was recovered by all pipelines except mothur (Figures S5 and S6).

In some cases, HTSA detected more known haplotypes (i.e., present in the database) than SSIZ. For example, two haplotypes of *B. violaceus* (Bv‐H1 and Bv‐H4) were detected by HTSA, with all pipelines and all sample types, in both jars from CON, where only one colony, and thus one haplotype, was associated to this species with SSIZ (jar A; unassigned colony, assigned later with *cyt b*; Figure 4a).

Haplotype distributions revealed by HTSA were always highly correlated to the one observed with SSIZ (r ranging from 0.932 to 0.965; Figure 4b,c, Figures S7‐S11). The lowest correlation was observed for 1‐year ebDNA processed with obitools (Figure 4c). A slight effect of the pipeline on correlation was detected (Friedman test; *χ* ^2^ = 11.462; *df* = 5; *p* = .043), but none of the pairwise comparisons were significant (Figure S12). Conversely, the type of sample had a strong effect (Friedman test; *χ* ^2^ = 16.4; *df* = 3; *p* < .001), with pairwise comparisons significant in most cases (Figure S12).

### 3.4 Population diversity indices

All H_S_ values, computed from HTSA for *B. diegensis* in each marina (Table S6), were positively correlated to those obtained from SSIZ, whatever the pipeline or the type of sample (r ranging from 0.668 to 0.935). One‐year ebDNA had consistently lower r values. An effect of the pipeline (Friedman test; *χ* ^2^ = 19.571; *df* = 5; *p* = .002) and of the type of sample (Friedman test; *χ* ^2^ = 16.2; *df* = 3; *p* = .001) was detected. However, none of the pairwise comparisons between pipelines were significant, even with dada2, which exhibited the highest correlation values (Figure S13). Conversely, all but one pairwise comparisons between the types of sample were significant (Figure S13).

**FIGURE 6:**
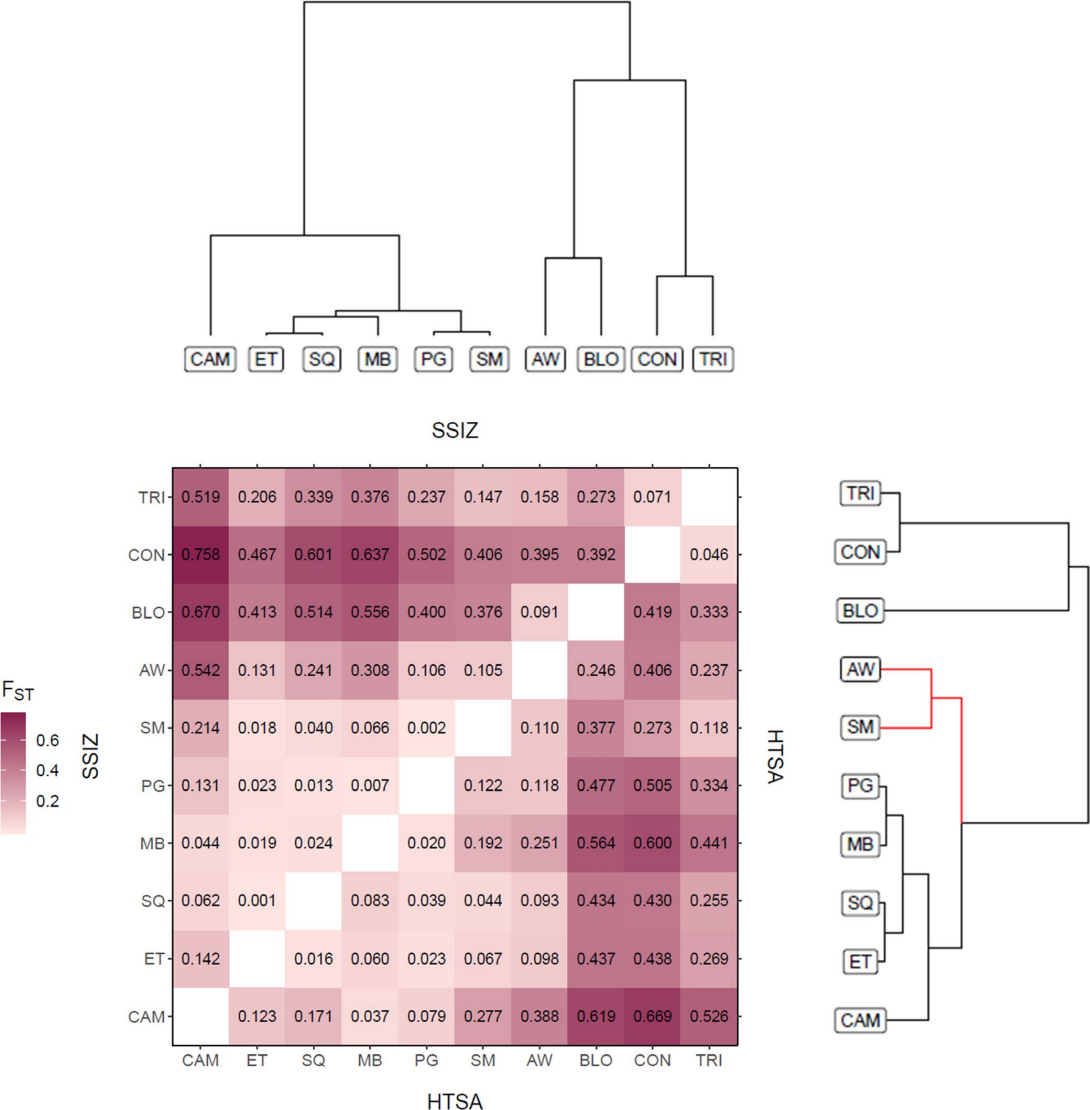
Pairwise F_ST_ values computed from SSIZ (top left) or HTSA (dada2, 3‐month ebDNA) (bottom right) data and population clustering based on pairwise F_ST_. The difference in clustering between the two datasets is highlighted in red. Location codes in Figure 1

Pairwise F_ST_ values obtained with SSIZ and HTSA data were highly correlated, and correlations were significant (Mantel test), whatever the pipeline or the type of sample (Table S7). Lower r values were always observed with 1‐year ebDNA. Clustering locations based on their pairwise F_ST_ led to similar results with both HTSA and SSIZ datasets, except for AW and SM (Figure 6).

## 4 DISCUSSION

We compared several bioinformatics pipelines to assess their ability to jointly analyze genetic (infraspecific) and taxonomic diversity, from high‐throughput sequencing (HTS) of DNA from preservative ethanol (ebDNA). Using mock communities, we evaluated the reliability of HTS as compared to Sanger haplotype sequencing carried out on the same assemblages. All tested pipelines were able to depict accurately both taxonomic and genetic diversity from ebDNA and bulkDNA datasets, showing the reliability of the HTS‐based approach. However, they performed differently regarding the balance between false positives and false negatives. Even if this experiment has been performed on a restricted taxonomic range, we believe that this approach can be successfully applied to a wide variety of organisms. In that sense, we highlight below the important issues to be taken into consideration for further studies.

### 4.1 Ethanol‐based DNA is a valid nondestructive alternative to bulkDNA, even after several months of storage

DNA from preservative ethanol has been used in a few metabarcoding studies on terrestrial or freshwater arthropods and fish (Zenker et al., 2020 and references therein). They showed that the amount of DNA released in ethanol differs depending on the taxa (Linard et al., 2016). Tunicates might be particularly challenging in that regard: zooids are embedded in a noncellular gelatinous tunic, composed of tunicin, which, like other polysaccharides, may decrease the amount and quality of DNA released in ethanol (Aboul‐Maaty & Oraby, 2019). Despite these particularities, we showed that ebDNA can be used to study marine invertebrates with HTSA, thus expanding its already known applicability to a wider range of organisms and environments.

The quality of 1‐year ebDNA seemed poorer, with lower correlations between HTSA and SSIZ (Figure 4c). DNA quantification was indeed impossible after 1‐year storage, and PCR amplifications were less efficient (several attempts have been made for every sample and no amplicon was obtained from ETA). These findings are congruent with those of Zenker et al. (2020) who had difficulties amplifying insect community DNA from preservative 98% ethanol after seven to 15 months. Because ebDNA allows to reuse the samples for other purposes (e.g., abundance estimation and morphological analyses), we recommend this approach for marine community analyses, preferably within 6 months after preservation. In the particular case of historical samples, the use of bulkDNA should be favored even if this implies the destruction of the samples.

### 4.2 Taxon‐targeted primers can improve the quantitative use of HTSA data

Population diversity indices are usually calculated from the frequency of individuals associated to each haplotype. With HTSA data, the proportion of reads of a particular haplotype is used as a proxy of its frequency in the population. However, several biases can occur during laboratory processing steps that can decrease the correlation between haplotype frequencies based on individual and read counts (Lamb et al., 2019). The amount of DNA released in ethanol can be highly variable depending on biomass and body composition (Marquina et al., 2019). In our case, all colonies were resized to approximately the same biomass. Another major source of bias is the primer annealing efficiency (Piñol et al., 2018), an issue that we circumvented by designing primers targeting the genus *Botrylloides* and encompassing the same diversity than with the primers used for SSIZ. Traditional primers such as those designed by Leray et al. (2013) amplifying a 313‐bp fragment would only have revealed four haplotypes in our dataset (two per species), thus decreasing the infraspecific polymorphism that could be examined. The choice of the marker length is a trade‐off between offering a sufficient infraspecific variability and being small enough to persist longer in ethanol. Moreover, the use of only one mitochondrial marker offers a limited view of the genetic diversity, and primers targeting specific taxa reduce information collected from a complex community. An alternative would be to use multiplexes of several primer pairs targeting multiple genomic regions and taxa (Corse et al., 2019). Additionally to the COI dataset, data from the HTS of 16S conducted on 6‐month ebDNA and bulkDNA, using the primers of Kelly et al. (2016), were analyzed (Appendix S1 [SI.6], Figure S2 and S3). Members of six phyla (mainly Bryozoa and Porifera) were identified, which most likely were epibionts or species embedded in the *Botrylloides*' tunic. These accompanying data on metazoan diversity of our assemblages showed that they were more complex than simple two‐species mock communities and provide support for the use of ebDNA with other primers and markers, either to study infraspecific diversity of a more diverse set of species or to jointly evaluate the overall taxonomic diversity of the assemblage.

### 4.3 Careful choice of bioinformatics pipeline is needed to examine genetic diversity

All tested pipelines described successfully the species composition and the overall genetic diversity of each community. However, they produced a high number of unexpected sequences, as reported in similar studies using other pipelines (Elbrecht et al., 2018; Stat et al., 2017). As a consequence, diversity indices based on haplotype counts (such as haplotype richness) are unreliable, similarly to species counts in taxonomic diversity studies (Calderón‐Sanou et al., 2020). Nonetheless, population genetic indices based on frequency data (H_S_, F_ST_) were correctly recovered because most spurious ASVs/OTUs accounted for only a small proportion of reads (1.5%–9%).

All pipelines produced results highly correlated to SSIZ. The correlation between haplotype distributions was highly influenced by sample types but only slightly by pipelines (Figure S12). Contrary to expectations, denoising‐based methods did not perform significantly better than clustering‐based approaches, when using high identity thresholds (Table S4; Appendix S1 [SI.5]). For instance, obitools (denoising–based) detected only five haplotypes, whereas seven were revealed with vsearch (clustering‐based). In pipelines that failed to retrieve some haplotypes, the missed ones were always removed at the denoising/clustering steps, except Bd‐H2, which was accurately clustered with vsearch‐ and swarm‐based pipelines but discarded at the index‐jump and replicate filtering steps. In such cases, the threshold chosen for post‐treatment filtering could be loosen but this would be at the expense of specificity with additional false positives. For example with vsearch, the OTU corresponding to Bd‐H2 is only represented by 1–6 reads per sample. Keeping it would require not to apply an index‐jump correction, which would lead to a total of 1,149 OTUs after the data processing steps.

Unexpected ASVs/OTUs that are slightly divergent from a haplotype might be either PCR or sequencing errors. Most PCR‐ and sequencing‐born unexpected sequences would have been discarded by our filtering step on PCR replicates (i.e., we retained only ASVs/OTUs present in at least 5 technical replicates), except if some errors occurred repeatedly because of particular sequence properties (e.g., mono‐ or dinucleotide repeats; Clarke et al., 2001). This points to the necessity of using tagged PCR replicates to detect false positives, as also suggested by Turon et al. (2020). The unexpected ASVs/OTUs might also be true haplotypes not identified by SSIZ because of chimerism (induced by colony fusion). Although not reported in the studied species, chimerism is documented in *Botrylloides niger* Herdman, 1886 colonies with a prevalence of 1.9% (Sheets et al., 2016). They might also come from small fragments of other colonies put accidentally into the jar. This could be the case for Bv‐H1 in CON, which has been reported in most samples collected in 2011 in CON (FV, unpublished data).

Sixteen ASVs/OTUs were highly divergent (<99% identity) from the known haplotypes and were easily identified as technical chimeras. The two pipelines including a chimera‐removal step successfully removed most of them (all for mothur, all but one for dada2), the others retained between 11 and 14 chimeras. Contrary to Tsuji, Miya, et al. (2020) who chose not to include a chimera‐removal step because of the high similarity between haplotypes, our results suggested that this step is crucial for limiting the number of unexpected ASVs/OTUs, without impairing the detection of true haplotypes.

### 4.4 Improving haplotype detection—a matter of compromise

Choosing an appropriate approach for read processing is a trade‐off between removing all technical errors and keeping all true sequences. The most sensitive pipelines, able to retrieve the highest number of haplotypes (dada2, vsearch), were also the ones producing the highest number of unexpected sequences. Results might be improved by fine‐tuning some of the parameters used (Appendix S1 [SI.5]). In all cases, however, the proportion of unexpected reads remains low and frequency‐based indices would only be slightly influenced by parameter choices.

Other approaches have been proposed to discriminate between errors and true sequences, such as LULU, which is based on sequence co‐occurrence in samples, or the protocol described in Turon et al. (2020), which is based on changes in the entropy (sensu Shannon entropy) ratio between the second and third codon positions. By processing the ASVs produced with dada2 with the LULU R package v‐0.1.0 (Frøslev et al., 2017), the number of false positives was lowered by 35%, but two rare true haplotypes were lost (Bd‐H7 and Bd‐H8). Index‐jump correction and replicate filtering thus appeared efficient enough to remove most PCR and sequencing errors, as suggested by Taberlet et al. (2012) or Tsuji, Miya, et al. (2020), removing 98.6% of unexpected sequences produced by dada2.

Overall, we showed that, when using community samples, ebDNA is a nondestructive alternative for a joint assessment of taxonomic and genetic diversity, thus expanding its applicability to a wider range of organisms and environments. The results detailed here, however, were obtained on a restricted set of organisms (two species of a given genus), and our conclusions might differ when considering other taxonomic groups. Some bioinformatics pipelines were able to discriminate between very similar haplotypes (with only 1 bp difference), which leads us to believe that the estimation of both inter‐ and intraspecific diversity would be effective for any kind of organism. In case of taxonomic groups in which cryptic species have been reported (i.e., taxa only recognized based on molecular data), the approach would also allow to identify them and describe the distribution of the genetic diversity within and among these lineages. Nonetheless, this approach requires that the targeted accepted species are evolutionarily divergent enough (i.e., species for which there is a clear barcoding gap) and polymorphic with the marker used. The choice of the marker and the primer design are thus key steps of the process and must be adapted to the targeted taxa for maximizing the possibility to identify either accepted species or cryptic lineages. So far, similar studies are rare, and we cannot ascertain if our results would remain similar with other case studies. We do feel, however, that the use of this HTS approach to reveal both taxonomic and genetic diversity should remain effective when used in a range of biological settings. This hypothesis needs to be confirmed by further investigations, for which we can give some recommendations based on our results: (a) using primer sets designed to target a genus or a family, if possible multiplexed to overcome limitations in taxonomic and genomic coverage, (b) using dada2 which includes a chimera‐removal step, and (c) using post‐treatment filters based on index‐jump correction and on PCR replicates filtering.

## CONFLICT OF INTEREST

None declared.

## AUTHOR CONTRIBUTIONS

**Marjorie Couton:** Data curation (lead); formal analysis (equal); investigation (equal); methodology (equal); software (lead); writing–original draft (equal); writing–review and editing (equal). **Aurélien Baud:** Formal analysis (equal); investigation (equal). **Claire Daguin‐Thiébaut:** Investigation (equal); methodology (equal); validation (equal); writing–review and editing (equal). **Erwan Corre:** Methodology (equal); resources (equal); writing–review and editing (equal). **Thierry Comtet:** Conceptualization (equal); investigation (equal); methodology (equal); supervision (equal); writing–original draft (equal); writing–review and editing (equal). **Frédérique Viard:** Conceptualization (equal); formal analysis (equal); funding acquisition (lead); investigation (equal); methodology (equal); supervision (equal); writing–original draft (equal); writing–review and editing (equal).

## Supporting information

Supplementary Material

## Data Availability

DNA sequences: GenBank accessions for two new *Botrylloides diegensis* haplotypes: MT586698 and MT586699; MiSeq sequence data on NCBI SRA: PRJNA639681 and PRJNA639923. Scripts for the six metabarcoding pipelines: https://github.com/joarwrie/HTS_pipelines‐for‐haplotype‐and‐species‐recovery
