# Supplementary material for: High‐throughput sequencing on preservative ethanol is effective at jointly examining infraspecific and taxonomic diversity, although bioinformatics pipelines do not perform equally
Source: Ecol Evol. 2021 Mar 23;11(10):5533–46. doi: 10.1002/ece3.7453 (PMC8131761; doi:10.1002/ece3.7453)
Supplement: Supplementary file 1 — Supplementary Material [file ECE3-11-5533-s001.docx]

# Supporting information

## SI.1 – Protocol for single zooids DNA extraction and amplification

One zooid from each colony was isolated in ethanol under a dissecting microscope. DNA was extracted using the NucleoSpin® Tissue 96-well Kit (Macherey-Nagel), following the manufacturer’s protocol with few modifications: just before lysis, each zooid was rinsed in PBS buffer (1X) to remove ethanol, dried on an absorbent paper, and placed in a 8-tube strip, containing buffer lysis T1 and proteinase K. After 2-3h at 56°C, an additional volume of 25µl of proteinase K (20 mg mL^-1^) was added to the lysis buffer and lysis was completed overnight. A two-step elution was performed with twice 60 µL of elution buffer pre-heated at 70°C. DNA extracts were stored at -20°C until amplification.

A 709-bp COI fragment was amplified for each zooid using the primers designed by Folmer, Black, Hoeh, Lutz, and Vrijenhoek (1994) LCO1490: 5’-GGTCAACAAATCATAAAGATATTGG-3’ and HCO2198: 5’- TAAACTTCAGGGTGACCAAAAAATCA-3’. For each reaction the total volume (25 µL) was composed of 0.5 U GoTaq® DNA polymerase (Promega), 1X reaction buffer, 50 µM dNTPs, 2 mM MgCl_2_, 48 ng µL^-1^ of bovine serum albumin (BSA), 0.3 µM of each primer and 5 µL of stock DNA. Amplification involved an initial denaturation step at 94 °C for 3 min, followed by 35 cycles at 94 °C for 50 s, 51.5 °C for 50 s and 72 °C for 1 min, and a final extension step at 72 °C for 5 min.

In order to improve amplification and sequence quality for 17 *Botrylloides violaceus* samples, and 59 *B. diegensis* samples, a second amplification and sequencing was done with specific primers, also targeting the Folmer region. For *B. violaceus*, we used primers designed by Callahan, Deibel, McKenzie, Hall, and Rise (2010) Violet_Forward: 5’-TTAGGTTTTGGTCTAGGTTTATTG-3’ and Violet_Reverse: 5’-TAAATGTTGATAAAGTACAGGGTC-3’, amplifying a 644-bp fragment. For *B. diegensis*, we used newly designed primers: Bdieg-COI-F: 5’-TGTCTACTAATCATAAAGATATTAG-3’ and Bdieg-COI-R2: 5’-AATATACACTTCAGGGTGTCCAA-3’, amplifying a fragment of 713 bp. For each reaction the total volume (25 µL) was composed of 1 U GoTaq® DNA polymerase (Promega), 1X reaction buffer, 0.2 mM dNTPs, 2 mM MgCl_2_, 12 ng µL^-1^ of BSA, 0.8 µM of each primer and 5µl of DNA stock solution. For the two specific markers, amplification involved an initial denaturation step at 94 °C for 3 min, followed by 35 cycles at 94 °C for 50 s, 49.5 °C for 50 s and 72 °C for 1 min, and a final extension step at 72 °C for 5 min.

PCR products were Sanger sequenced in both directions by Eurofins Genomics (Germany, GmbH). Sequences were aligned using CodonCode Aligner v.5.0.1 (CodonCode Corporation, Dedham, MA), and trimmed to 607-bp for *B. diegensis* and 580-bp for *B. violaceus*.

## SI.2 – Protocol for DNA extraction from preservative ethanol and communities

During all extraction procedures, precautions were taken to avoid contamination of DNA extracts with external DNA: all consumables not sold as DNA-free were immersed in 12.5 % commercial bleach (0.65 % hypochlorite) for at least 30 min, rinsed and placed under UV light for at least 15 min. All equipment and bench surfaces were also DNA decontaminated before use.

After 3, 6, and 12 months of storage, each jar was shaken by several inversions, and three replicates of 1 mL preservative ethanol were pipetted and deposited in a deep-well plate. This plate was then placed in a dry block at 70 °C, under a fume hood overnight to allow the ethanol to evaporate. The DNA was then purified using the NucleoSpin® Tissue 96-well Kit (Macherey-Nagel). For lysis, 200 µL of T1 buffer and 25 µL of proteinase K (20 mg.mL^-1^) were added in each well and the plate was incubated at 56°C for 15 min. After that, the manufacturer’s protocol was followed until the end. Elution was performed in 30 µL of elution buffer pre-heated at 70°C. The volume gathered after the first elution was placed again on the column for a second elution to maximize the yield. One extraction control was performed for each storage duration by adding lysis buffer on an empty well of the extraction plate, and they were treated as the other samples for all further steps of the process. DNA concentrations were quantified by fluorescence using a PicoGreen® Quant-It^TM^ dsDNA kit. DNA samples were stored at -20°C.

Soon after the collection of the last preservative ethanol sample (after one year of storage), all the material (i.e. colonies and remaining ethanol) from a jar was transferred to a 2-L glass beaker, mixed with an immersion blender (ErgoMixx MSM66020, BOSCH®) until homogenization, and then filtered with a 41-µm mesh size nylon filter. The solid fraction (> 41 µm) was stored in a 50 mL tube at -20 °C until DNA extraction, which was performed no longer than four weeks later. DNA extraction was performed using the NucleoSpin® Soil kit (Macherey-Nagel). Previous to lysis, 300 mg of wet material of each sample were put in an oven at 70 °C for 10 min to allow evaporation of residual ethanol. The bead beating step from the manufacturer’s protocol was replaced by an overnight lysis step at 56 °C where 1 mL of SL1 buffer, 150 µL of SX buffer and 30 µL of proteinase K (20 mg.mL^-1^) were added to each tube. The subsequent steps were performed following the manufacturer’s protocol. Each sample was extracted in three replicates and two extraction controls were performed by adding lysis buffer to an empty tube and by performing all further extraction steps as for the other samples. DNA was quantified by absorbance in a Spark TECAN reader using a NanoQuant Plate^TM^. DNA samples were stored at -20°C.

## SI.3 – Design of primers targeting *Botrylloides* spp. for HTS

A wide variety of primers, targeting broad taxonomic ranges, have been designed for metabarcoding purposes on various markers. Such primers are known, however, to exhibit amplification bias due to primer mismatches with the sequences of various species (Collins et al., 2019). One example for COI is the primer pair designed by Leray et al. (2013), for which bias has been shown when amplifying ascidians (e.g. Couton, Comtet, Le Cam, Corre, & Viard, 2019). Such an issue might cause the non-detection of a particular species or lineage in a mixture of DNAs, or bias the relative proportion of DNA amplified for a given taxon. To circumvent this problem, a novel set of primers was designed to amplify preferentially species from the genus *Botrylloides*, and to exclude the closely related genus *Botryllus*; *Botryllus schlosseri* being another colonial ascidian conspicuous in the study locations. Moreover, the targeted fragment was chosen to capture the same genetic diversity as the COI fragment amplified by the primers designed by Folmer et al. (1994), which are commonly used for barcoding *Botrylloides* species, while being shorter to allow sequencing on an Illumina platform. In particular, we aimed at recovering all known haplotypes in the targeted species (Viard et al., 2019; this study).

The newly designed primers were tested *in silico* with ecoPCR from the obitools-1.2.11 (Boyer et al., 2016) against the NCBI nucleotide database (nt). When considering only sequences with a maximum of three mismatches with primer sequences, eight species were recovered with an appropriate fragment length, all being ascidians (Table S1).

**Table S1** List of species recovered *via* *in silico* PCR using ecoPCR against the NCBI nucleotide database (nt) with the newly designed primers COIBotrF2.2 – 5’-AGTGTTTTYATTCGTWTAGA-3’, and COIBotrR7.1 – 5’-CAAAACARAGAYATRGARAAYAT-3’. A maximum of three mismatches per primer was allowed. The number of sequences retrieved (# seq), the number of minimum forward (min F mm) and reverse (min R mm) mismatches, and the length of the fragment (primers excluded) is given for each species.

| Species | Family | Phylum | # seq | min F mm | min R mm | Length |
| --- | --- | --- | --- | --- | --- | --- |
| *Botrylloides violaceus* | Styelidae | Chordata | 18 | 0 | 0 | 412 |
| *Botrylloides diegensis* | Styelidae | Chordata | 3 | 0 | 0 | 412 |
| *Botrylloides fuscus* | Styelidae | Chordata | 1 | 0 | 2 | 412 |
| *Botrylloides nigrum* | Styelidae | Chordata | 1 | 3 | 2 | 412 |
| *Didemnum vexillum* | Didemnidae | Chordata | 2 | 2 | 3 | 412 |
| *Didemnum* sp. | Didemnidae | Chordata | 2 | 3 | 3 | 412 |
| *Pyura praeputialis* | Pyuridae | Chordata | 1 | 2 | 1 | 412 |
| *Pyura* sp. | Pyuridae | Chordata | 36 | 2 | 2 | 412 |
| *Caligus rogercresseyi* | Caligidae | Arthropoda | 1 | 2 | 3 | 825 |

Four of these species belong to the genus *Botrylloides*, two of them being expected in our dataset (*B. violaceus* and *B. diegensis*), and showing no primer mismatches. The third species present in our study area (*B. leachii*) was not recovered because no sequence was present in the database with both priming sites. Four other ascidian species/taxa were recovered, with the correct sequence length, from two genera and two families, but with at least one mismatch on each priming site. Finally, one arthropod species was recovered but it corresponds to nuclear DNA (chromosome 8) and has a fragment length two times higher than expected.

The primers were then tested on template DNA from the three *Botrylloides* species potentially present in our study area, covering all known local haplotype diversity, as well as from other ascidian species, belonging to other families but found in the study locations, including *Botryllus schlosseri*. The results did not reveal any amplification bias for any of the tested haplotypes, and showed no amplification for the non-targeted taxa (Fig. S1).


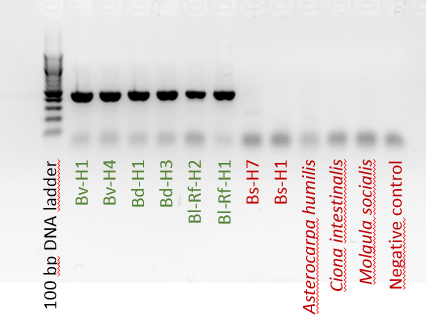


**Figure S1** Amplification efficiency of the primer pair designed in this study (COIBotrF2.2/COIBotrR7.1) on six haplotypes from three species of the targeted genus (*Botrylloides violaceus*: Bv; *B. diegensis*: Bd; *B. leachii*: Bl), two haplotypes from the closely related species *Botryllus schlosseri* (Bs) and three other ascidian species. The last well contains a PCR negative control.

## SI.4 – Protocol for the two-step PCR COI library preparation

All library preparation steps were performed in the respect of strict rules preventing contamination of samples. They include, for example, the UV irradiation of all tips, plates and tubes before use, the mandatory use of filter tips, the strict separation of pre- and post-PCR steps in different labs.

Library preparation was performed using a dual-barcoded, dual-indexed two-step PCR procedure. First, a 455-bp COI portion was amplified for each sample using primers specifically designed to target species from the genus *Botrylloides* (see details in SI.3): COIBotrF2.2 - 5’-AGTGTTTTYATTCGTWTAGA-3’ and COIBotrR7.1 - 5’-CAAAACARAGAYATRGARAAYAT-3’. At the 5’ end of each primer, a Nextera tail and an 8-bp tag were added to identify PCR and extraction replicates (see Table S2). Nine replicates (three tagged PCR pools for each of the three extraction replicates) were identified using a unique combination of five different tags.

**Table S2** Sequences of Nextera-tailed and tagged primers for amplifying COI in ebDNA and bulkDNA samples. The tag sequence is in bold. Stars indicate phosphorothiate bounds protecting primers from 3’ exonuclease activity of the Q5 enzyme.

| NXTtag1_COIBotrF2.2 | TCGTCGGCAGCGTCAGATGTGTATAAGAGACAG**AGTATGCC**AGTGTTTTYATTCGTWTA*G*A |
| --- | --- |
| NXTtag2_COIBotrF2.2 | TCGTCGGCAGCGTCAGATGTGTATAAGAGACAG**CTGGACTA**AGTGTTTTYATTCGTWTA*G*A |
| NXTtag3_COIBotrF2.2 | TCGTCGGCAGCGTCAGATGTGTATAAGAGACAG**GCACGTAT**AGTGTTTTYATTCGTWTA*G*A |
| NXTtag4_COIBotrF2.2 | TCGTCGGCAGCGTCAGATGTGTATAAGAGACAG**TACTCAGG**AGTGTTTTYATTCGTWTA*G*A |
| NXTtag5_COIBotrF2.2 | TCGTCGGCAGCGTCAGATGTGTATAAGAGACAG**ATCTTCAG**AGTGTTTTYATTCGTWTA*G*A |
| NXTtag1_COIBotrR7.1 | GTCTCGTGGGCTCGGAGATGTGTATAAGAGACAG**AGTATGCC**CAAAACARAGAYATRGARAAY*A*T |
| NXTtag2_COIBotrR7.1 | GTCTCGTGGGCTCGGAGATGTGTATAAGAGACAG**CTGGACTA**CAAAACARAGAYATRGARAAY*A*T |
| NXTtag3_COIBotrR7.1 | GTCTCGTGGGCTCGGAGATGTGTATAAGAGACAG**GCACGTAT**CAAAACARAGAYATRGARAAY*A*T |
| NXTtag4_COIBotrR7.1 | GTCTCGTGGGCTCGGAGATGTGTATAAGAGACAG**TACTCAGG**CAAAACARAGAYATRGARAAY*A*T |
| NXTtag5_COIBotrR7.1 | GTCTCGTGGGCTCGGAGATGTGTATAAGAGACAG**ATCTTCAG**CAAAACARAGAYATRGARAAY*A*T |

Each PCR reaction was performed in a total volume of 10 µL, composed of 0.3 U of Q5® Hot Start High-Fidelity DNA polymerase (New England Biolabs®, Inc.), 1X reaction buffer, 160 µM dNTPs, 0.3 µM of each tagged primer, 0.3 µM of the same primers without the tag and the Illumina tail (to enhance amplification), and 2 ng DNA template. Amplification involved an initial denaturation step at 98 °C for 4 min, followed by 35 cycles at 94 °C for 1 min, 46 °C for 45 s and 72 °C for 1 min, and a final extension step at 72 °C for 10 min. PCR products were checked on a 1.5% agarose gel and visualised under UV light after ethidium bromide staining. For each extraction replicate, three tagged-primer combinations were used. To reduce any stochastic biases that could appear during amplification, three independent PCRs, using three different thermocyclers, were performed with each tagged-primer combination. The three PCR products amplified with the same tagged-primer combination were pooled. A total of nine technical replicates was thus obtained (i.e. three tagged-PCR replicates for each of the three extraction replicates per sample). After this first PCR step, the three tagged replicates from the same DNA extraction sample were pooled according to their intensity on the agarose gel visualisation. All pools were purified with paramagnetic beads in order to remove excess primers and putative primer dimers using the NucleoMag® NGS clean up and size select kit following the manufacturer’s protocol (ratio of 1:1 PCR product vs. beads).

A second PCR was performed to complete Illumina® adapters and insert an index allowing sample identification (a sample being a combination of a jar and a type of sample; Fig. 2). We used the list of indexed adapters described in the Illumina® Nextera XT library preparation protocol (8 indexes i5 and 12 i7 allowing 96 combinations, see Table S3). PCR reactions were performed in a total volume of 10 µL composed of 0.4 U of Q5® Hotstart High-Fidelity DNA polymerase (New England Biolabs®, Inc.), 1X reaction buffer, 200 µM dNTPs, 0.13 µM of each primer and 1 µL DNA template. Amplification involved an initial denaturation step at 98 °C for 4 min, followed by 12 cycles at 98 °C for 30 s, 55 °C for 30 s and 72 °C for 30 s, and a final extension step at 72 °C for 5 min.

**Table S3** Nextera XT i5 and i7 indices used in combination in the second PCR. i5 and i7 indices are in bold; stars indicate phosphorothiate bounds protecting primers from 3’ exonuclease activity of the Q5 enzyme.

| i7_N701 | CAAGCAGAAGACGGCATACGAGAT**TCGCCTTA**GTCTCGTGGGCTC*G*G |
| --- | --- |
| i7_N702 | CAAGCAGAAGACGGCATACGAGAT**CTAGTACG**GTCTCGTGGGCTC*G*G |
| i7_N703 | CAAGCAGAAGACGGCATACGAGAT**TTCTGCCT**GTCTCGTGGGCTC*G*G |
| i7_N705 | CAAGCAGAAGACGGCATACGAGAT**AGGAGTCC**GTCTCGTGGGCTC*G*G |
| i7_N706 | CAAGCAGAAGACGGCATACGAGAT**CATGCCTA**GTCTCGTGGGCTC*G*G |
| i7_N707 | CAAGCAGAAGACGGCATACGAGAT**GTAGAGAG**GTCTCGTGGGCTC*G*G |
| i7_N710 | CAAGCAGAAGACGGCATACGAGAT**CAGCCTCG**GTCTCGTGGGCTC*G*G |
| i7_N711 | CAAGCAGAAGACGGCATACGAGAT**TGCCTCTT**GTCTCGTGGGCTC*G*G |
| i7_N712 | CAAGCAGAAGACGGCATACGAGAT**TCCTCTAC**GTCTCGTGGGCTC*G*G |
| i7_N714 | CAAGCAGAAGACGGCATACGAGAT**TCATGAGC**GTCTCGTGGGCTC*G*G |
| i7_N720 | CAAGCAGAAGACGGCATACGAGAT**AGGCTCCG**GTCTCGTGGGCTC*G*G |
| i7_N723 | CAAGCAGAAGACGGCATACGAGAT**GAGCGCTA**GTCTCGTGGGCTC*G*G |
| i5_S503 | AATGATACGGCGACCACCGAGATCTACAC**TATCCTCT**TCGTCGGCAGCG*T*C |
| i5_S505 | AATGATACGGCGACCACCGAGATCTACAC**GTAAGGAG**TCGTCGGCAGCG*T*C |
| i5_S506 | AATGATACGGCGACCACCGAGATCTACAC**ACTGCATA**TCGTCGGCAGCG*T*C |
| i5_S510 | AATGATACGGCGACCACCGAGATCTACAC**CGTCTAAT**TCGTCGGCAGCG*T*C |
| i5_S513 | AATGATACGGCGACCACCGAGATCTACAC**TCGACTAG**TCGTCGGCAGCG*T*C |
| i5_S516 | AATGATACGGCGACCACCGAGATCTACAC**CCTAGAGT**TCGTCGGCAGCG*T*C |
| i5_S517 | AATGATACGGCGACCACCGAGATCTACAC**GCGTAAGA**TCGTCGGCAGCG*T*C |
| i5_S522 | AATGATACGGCGACCACCGAGATCTACAC**TTATGCGA**TCGTCGGCAGCG*T*C |

After the second PCR, all PCR products were checked on a 1.5% agarose gel and vizualized under UV light after ethidium bromide staining. The samples were then pooled according to their intensity on the agarose gel visualisation and the pool was purified by paramagnetic beads using the NucleoMag® NGS clean up and size select kit following manufacturer’s protocol (ratio 1:1 PCR products vs. beads). Quantification of the library was performed with qPCR with the NEBNext®Library Quant Kit for Illumina® (New England Biolabs®, Inc.) and a DNA profile was performed using an DNA 1000 chip in a 1200 Bioanalyzer equipment (Agilent technologies, Inc.). Sequencing was performed on an Illumina® MiSeq sequencer with a 600 cycles v3 protocol with two index reads.

## SI.5 – Effect of parameter values on the amount of false negatives and false positives

For each pipeline, the use of different parameter values might have an impact on the number of expected haplotypes that are not recovered (false negatives) and the number of errors kept in the final dataset (false positives). Choosing *a priori* the most appropriate parameter values is difficult. In that sense, tools developers usually test extensively all parameters and set the values they found out to be the most effective for most datasets as default. They can also recommend different sets of values depending on the question to be addressed.

Here, we compared pipelines with all parameter values set to default, except for those defining clustering/denoising algorithms. For these, we chose values that were described, or previously tested, as the most sensitive (i.e. able to retrieve rare sequences) and which would allow us to distinguish between closely related sequences (i.e. haplotypes within species). This blind choice allowed us to evaluate the effectiveness of each pipeline as it is most commonly used (i.e. without performing a test on mock communities beforehand). Results presented in the main text correspond to these sets of parameter values, and are in bold in Table S4 below. However, different clustering/denoising parameter values were also tested to evaluate their impact on the amount of false positives and false negatives retrieved from our dataset. Results are summarized in Table S4.

For denoising algorithms, the number of expected haplotypes detected did not (dada2), or slightly (obitools), vary depending on the parameter values used at the denoising step. Less sensitive approaches, however, revealed less false positives which accounted for a lower proportion of the total amount of reads. This was expected since denoising algorithms remove sequences from the dataset, hence decreasing the proportion of reads attributed to errors. It is important to note, however, that the sequencing coverage for our dataset was very high, and that less sensitive parameter values might have missed some expected haplotypes if the sequencing coverage had been reduced. For clustering-based tools, the number of haplotypes retrieved by the different sets of parameter values varied more drastically (Table S4). For example, seven expected haplotypes instead of three were identified with the most sensitive values as compared to the default values for vsearch. The number of false positives, however, is also higher for more sensitive values, but they do not necessarily represent a higher proportion of the total number of reads for clustering methods. Indeed these approaches, contrarily to denoising-based tools, which remove reads from the final dataset, keep the same total number of reads after processing, whatever the parameter value. In this case, the proportion of reads associated to false positives can vary depending on the way they have been grouped in OTUs.

Initially chosen parameter values that were used for comparisons presented in the main text (Table 1 corresponding to the bold lines in Table S4), were always the one allowing to retrieve the highest number of haplotypes (except for obitools). For clustering-based algorithms with fixed identity thresholds, the default parameter (97%) is commonly used to group sequences in OTUs representative of taxonomic (species) diversity. It was thus expected that choosing a higher identity threshold (99.5%) would increase the number of OTUs produced, and consequently increase our ability to recover expected haplotypes. This is congruent with our observations, as exemplified by vsearch which only identified three haplotypes when used with default parameters, two for *B. diegensis* and one for *B. violaceus.* However, when increasing the identity threshold for clustering, the number of false negatives decreased, and was not always accompanied by an increase in false positives.

In conclusion, with regular datasets (i.e. in the absence of mock assemblages) and the objectives of jointly examining taxonomic and genetic diversity, we recommend to use the set of parameter values that is described by the developers as the most sensitive for each tool, and especially for clustering algorithms. Tools based on denoising are less impacted by the different parameter values and the choice of using a more stringent set of values can be applied if the analyzed dataset has a sufficient sequencing coverage. Exploring these various sets of parameter values also confirmed our recommendation to use dada2, which is the only one to recover all expected haplotypes (across different sets of parameter values).

**Table S4** Number of ASVs/OTUs retrieved with different parameter values for the six pipelines, and retained after post-treatment corrections (index-jump and selection on replicates). After comparison with SSIZ results, the number of expected haplotypes recovered, the names of missing haplotypes, and the proportion of reads associated with unexpected sequences are indicated. Values in bold are those used in the main text (see Table 1). The reason for which these particular values were tested is indicated in the third column.

| Pipeline | Parameter value | Reason for choice | ASVs/OTUs | Index-jump correction | Present in at least five replicates | Expected haplotypes recovered | Missing haplotypes | % reads of unexpected sequences |
| --- | --- | --- | --- | --- | --- | --- | --- | --- |
| DADA2 | **pool=True** | **Most sensitive** | **2115** | **58** | **29** | **9** | **-** | **9** |
|  | pool=False | Default value | 368 | 85 | 17 | 9 | - | 0.7 |
|  | pool="pseudo" | Intermediate between the two others | 502 | 129 | 27 | 9 | - | 7.8 |
| OBITOOLS | **d=2, r=0.025** | **Sensitive in detecting rare species ^a^** | **4062** | **46** | **23** | **5** | **Bd-H2; Bd-H5; Bd-H7; Bd-H8** | **5** |
|  | d=1, r=1 | Default values | 1108 | 38 | 18 | 5 | Bd-H2; Bd-H5; Bd-H7; Bd-H8 | 1.1 |
|  | d=1, r=0.5 | Effective in another study ^b^ | 1504 | 44 | 22 | 6 | Bd-H2; Bd-H5; Bd-H8 | 1.1 |
| OBI+SWARM | **d=1, f** | **Most sensitive, recommended by authors** | **896** | **46** | **23** | **5** | **Bd-H2; Bd-H5; Bd-H7; Bd-H8** | **3** |
|  | d=2 | Other possibility recommended by authors | 237 | 28 | 16 | 4 | Bd-H2; Bd-H5; Bd-H6; Bd-H7; Bd-H8 | 1.3 |
|  | d=3 | Other possibility recommended by authors | 111 | 14 | 12 | 3 | Bd-H2; Bd-H3; Bd-H5; Bd-H6; Bd-H7; Bd-H8 | 1 |
| VS+SWARM | **d=1, f** | **Most sensitive,**  **recommended by authors** | **1386** | **46** | **22** | **5** | **Bd-H2; Bd-H5; Bd-H7; Bd-H8** | **1.5** |
|  | d=2 | Other possibility recommended by authors | 465 | 20 | 20 | 4 | Bd-H2; Bd-H5; Bd-H6; Bd-H7; Bd-H8 | 3.8 |
|  | d=3 | Other possibility recommended by authors | 234 | 13 | 13 | 3 | Bd-H2; Bd-H3; Bd-H5; Bd-H6; Bd-H7; Bd-H8 | 3.6 |
| VSEARCH | **99.50% id** | **High discriminating power for similar sequences** | **3055** | **64** | **36** | **7** | **Bd-H2; Bd-H8** | **8** |
|  | 97% id | Default value | 80 | 17 | 12 | 3 | Bd-H2; Bd-H5; Bd-H6; Bd-H7; Bd-H8; Bv-H1 | 4.8 |
|  | 99% id | Other | 658 | 75 | 41 | 5 | Bd-H2; Bd-H5; Bd-H8; Bv-H1 | 18.7 |
| MOTHUR | **99.50% id** | **High discriminating power for similar sequences** | **3270** | **34** | **20** | **6** | **Bd-H2; Bd-H5; Bd-H8** | **2** |
|  | 99% id | Other | 596 | 10 | 7 | 4 | Bd-H2; Bd-H3; Bd-H5; Bd-H7; Bd-H8 | 1.8 |
|  | 98.50% id | Other | 198 | 4 | 3 | 2 | Bd-H2; Bd-H5; Bd-H6; Bd-H7; Bd-H8; Bv-H1; Bv-h4 | 11.3 |

^a^ see Couton et al. (2019) for details.

^b^ see Calderón-Sanou, Münkemüller, Boyer, Zinger, and Thuiller (2020)

## SI.6 – Community analysis based on 16S: protocol and results

In order to evaluate the overall diversity of metazoan species found within each jar, besides the targeted *Botrylloides* species, which includes the species associated to the sampled colonies (e.g. epibionts), a fragment of the 16S rDNA gene was amplified, for the 6-month ebDNA and the bulkDNA extracts.

Library preparation and quantification were performed following the same two-steps PCR protocol as for COI, which is detailed in the Supporting Information SI.4 above. Amplifications were done using the primer set designed by Kelly et al. (2016): 16S_Metazoa_fwd – 5’-AGTTACYYTAGGGATAACAGCG-3’ and 16S_Metazoa_rev – 5’-CCGGTCTGAACTCAGATCAYGT-3’. The first PCR reaction (10 µL total volume) was composed of 0.6 U of Q5® High-Fidelity DNA polymerase (New England Biolabs®, Inc.), 1X reaction buffer, 200 µM dNTPs, 0.67 µM of each primer and 2 ng DNA template. Amplification involved an initial denaturation step at 98 °C for 4 min, followed by 40 cycles at 94 °C for 50 s, 61 °C for 45 s and 72 °C for 50 s, and a final extension step at 72 °C for 10 min. Sequencing was performed on an Illumina® MiSeq sequencer with a 300 cycles v2 micro cassette.

The 16S HTSA dataset was processed using dada2 v-1.13.1 (Callahan et al., 2016), a denoising algorithm which removes PCR and sequencing errors and produces a set of amplicon sequence variants (ASVs). Index-jumping and replicate filters were applied as for the COI HTS dataset. ASVs resulting from the dada2 analysis of the 16S dataset were then assigned against the nt GenBank database using the ecotag command from the obitools package, with no minimum identity threshold.

Over all samples, 492,707 reads were obtained corresponding to 135 ASVs. Taxonomic assignment revealed that 99.2% of the reads corresponded to metazoans, as expected when using the 16S_Metazoa primer set, designed by Kelly et al. (2016) to exclude non-metazoan taxa. The other 0.8% were unassigned eukaryotes. The most represented metazoan phyla were Bryozoa (76.5%), Porifera (13.4%), and Echinodermata (5.5%). The remaining 4.6% were assigned to Arthropoda, Nemertea, Mollusca, and unidentified metazoans (Fig. S2).


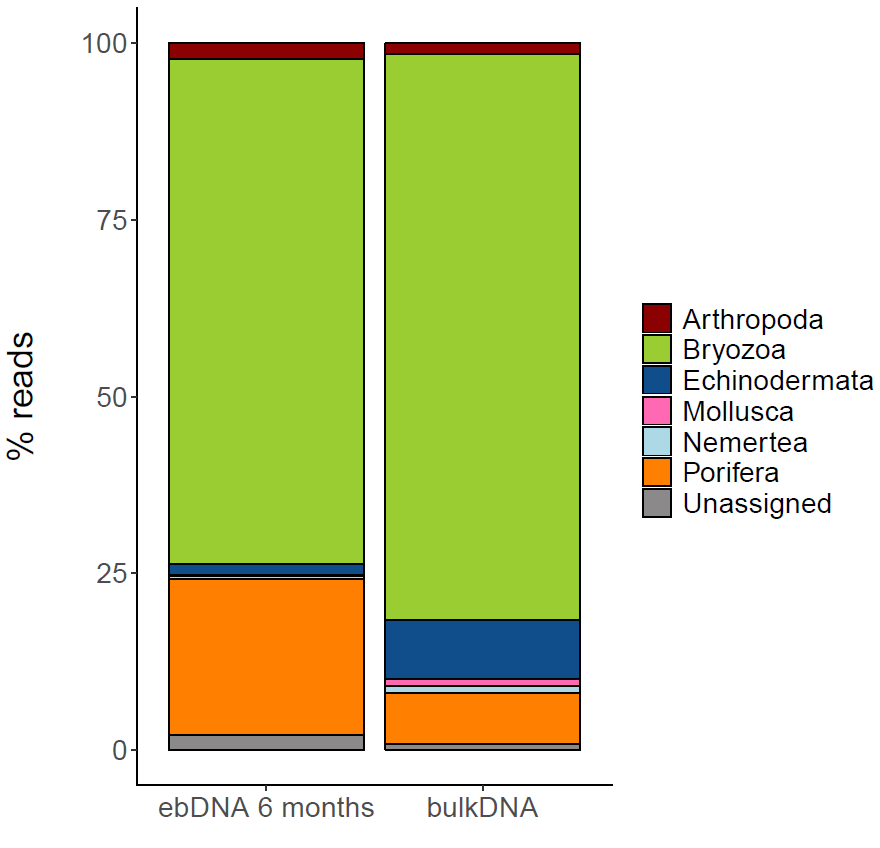


**Figure S2** Proportion of 16S reads assigned to each listed phylum for ethanol-based DNA (ebDNA 6 months) and bulkDNA samples using the ecotag tool.

Importantly, no tunicates were found in our samples, which is surprising since we mostly expected DNA from tunicate species, notably *Botrylloides* spp. (in particular for bulkDNA) because they represented the majority of the biomass sampled. This result suggested important amplification biases for tunicates using Kelly et al. (2016)’s primers. PCR amplifications of DNA from *B. violaceus* individual zooids always resulted in faint bands when using these primers. This supports the existence of 16S amplification biases for the target species, which could moreover be exacerbated under competition with other DNA. These biases could be explained when looking at the priming sites: two complete mitochondrial genomes are available in Genbank for *B. violaceus* (accession no. HF548552) and *B. diegensis* (accession no. NC 024103; registered under *B. leachii* but shown to be *B. diegensis*, Viard et al., 2019), showing two (*B. violaceus*) and three (for *B. diegensis*) mismatches on the forward primer and five on the reverse primer (for both species).

Altogether 11 taxa were assigned down to the species or genus level (Fig. S3). The three most represented taxa, representing 65% of all reads, were *Watersipora subatra* (Ortmann, 1890) (34%), *Scrupocellaria maderensis* (now accepted as *Scrupocaberea maderensis* (Busk 1860)) (19%) and *Bugulina stolonifera* (Ryland, 1960) *(12%)*.


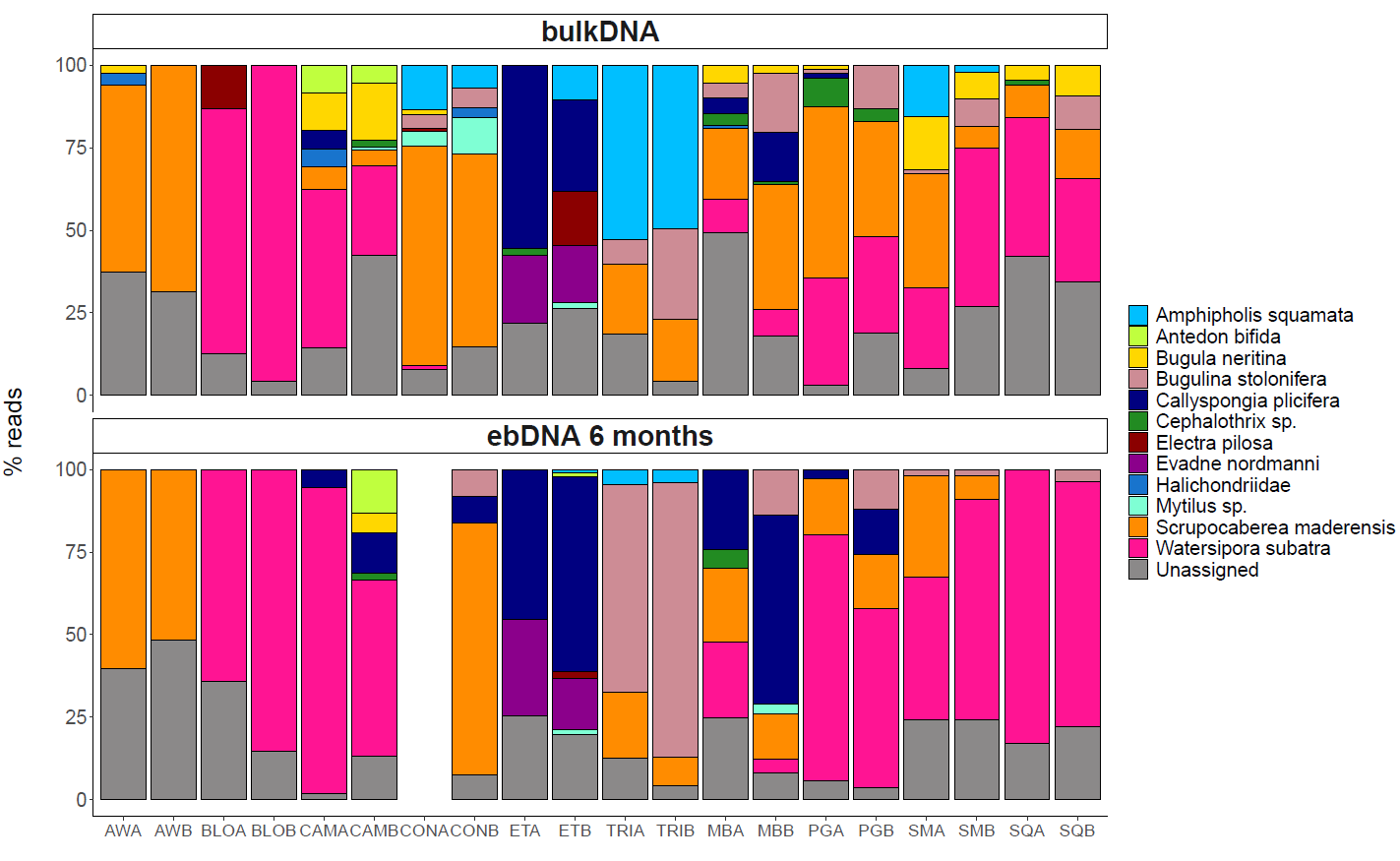


**Figure S3** Proportion of 16S reads assigned to each listed species, genus or family for ethanol-based DNA (ebDNA 6 months) and bulkDNA samples using the ecotag tool. The sample of 6-month ebDNA for jar A in Concarneau (CON) could not be amplified.

Interestingly, *W. subatra,* an introduced encrusting bryozoan, was found in all study ports in quadrats scrapped under pontoons, during the same field campaign as for the collection of the *Botrylloides* samples. Similarly, the introduced erected bryozoan *B. stolonifera*, although not conspicuous, has been regularly reported in several of our study ports, during Rapid Assessment Surveys carried out by the Station Biologique of Roscoff (F. Viard & L. Lévêque, unpublished data). Regarding *S. maderensis*, we believe that this is an assignment error, as only two *Scrupocellaria* species (and none under the genus name *Scrupocaberea)* are present in Genbank, namely *S. madarensis* and *S. varians* – now accepted as *Pomocellaria varians* (Hincks, 1882)). This does not include the indigenous *S. scruposa,* which is found in our port surveys. The 16S marker used seems to amplify effectively bryozoans, which are important epibionts of our target species, and conspicuous in ports. This effectiveness is however balanced by the low number of references available in public databases for this marker, an issue frequently encountered particularly for NIS, and deserving further work between taxonomists and molecular biologists (Darling et al., 2017).

**Table S5** Odds ratio (and p-values in parentheses) from Fisher’s exact tests calculated between the number of colonies assigned to each *Botrylloides* species with Sanger sequencing on individual zooid (SSIZ; expected values) and the number of reads assigned to the same species with COI high-throughput sequencing on assemblages (HTSA; observed values). Comparisons have been performed for each pipeline and each type of sample (ethanol-based DNA after 3 months, 6 months or 1 year, and bulk DNA) in the three locations where both species have been detected. Significant values are in bold according to the cut-off which controls the false discovery rate (0.004) calculated with the brainwaver v-1.6 R package following Benjamini and Yekutieli (2001).

| **Marina** | **Date** | **dada2** | **obitools** | **vsearch** | **obi+swarm** | **vs+swarm** | **mothur** |
| --- | --- | --- | --- | --- | --- | --- | --- |
| **AW** | **3m** | 0.701 (0.339) | 0.668 (0.250) | 0.686 (0.334) | 0.674 (0.332) | 0.700 (0.339) | 0.678 (0.333) |
|  | **6m** | 0.866 (0.715) | 0.837 (0.585) | 0.851 (0.713) | 0.830 (0.583) | 0.862 (0.714) | 0.850 (0.713) |
|  | **1y** | 0.897 (0.855) | 0.904 (0.856) | 0.886 (0.718) | 0.856 (0.713) | 0.899 (0.855) | 0.927 (0.857) |
|  | **bulk** | 0.758 (0.453) | 0.741 (0.449) | 0.752 (0.451) | 0.733 (0.447) | 0.764 (0.455) | 0.775 (0.458) |
| **PG** | **3m** | **0.166 (<0.001)** | **0.159 (<0.001)** | **0.162 (<0.001)** | **0.161 (<0.001)** | **0.170 (<0.001)** | **0.183 (<0.001)** |
|  | **6m** | **0.230 (0.001)** | **0.216 (<0.001)** | **0.230 (0.001)** | **0.230 (0.001)** | **0.243 (0.001)** | **0.255 (0.002)** |
|  | **1y** | **0.183 (<0.001)** | **0.180 (<0.001)** | **0.192 (<0.001)** | **0.194 (<0.001)** | **0.205 (<0.001)** | **0.206 (<0.001)** |
|  | **bulk** | 0.379 (0.039) | 0.366 (0.038) | 0.360 (0.038) | 0.351 (0.025) | 0.371 (0.038) | 0.424 (0.083) |
| **CON** | **3m** | **0.102 (0.002)** | **0.105 (0.004)** | **0.096 (0.002)** | **0.091 (0.001)** | **0.094 (0.001)** | 0.112 (0.006) |
|  | **6m** | **0.100 (0.002)** | 0.116 (0.006) | 0.115 (0.006) | **0.110 (0.004)** | 0.113 (0.006) | 0.120 (0.009) |
|  | **1y** | **0.093 (0.001)** | **0.101 (0.002)** | **0.099 (0.002)** | **0.095 (0.001)** | **0.099 (0.002)** | **0.106 (0.003)** |
|  | **bulk** | **0.070 (<0.001)** | **0.074 (<0.001)** | **0.066 (<0.001)** | **0.063 (<0.001)** | **0.066 (<0.001)** | **0.080 (<0.001)** |

**Table S6** Values of average gene diversity per locus (Hs; as described by Nei (1973)) computed from the number of colonies (SSIZ) or the abundance of ASVs/OTUs (HTSA) for each marina, per pipeline and sample type (ebDNA after 3 months, 6 months or 1 year of storage and bulk DNA). See Fig. 1 for the codes of the marinas.

|  |  | **dada2** | | | | **obitools** | | | | **vsearch** | | | | **obi+swarm** | | | | **vs+swarm** | | | | **mothur** | | | |
| --- | --- | --- | --- | --- | --- | --- | --- | --- | --- | --- | --- | --- | --- | --- | --- | --- | --- | --- | --- | --- | --- | --- | --- | --- | --- |
| **Location** | SSIZ | 3mo | 6mo | 1yr | bulk | 3mo | 6mo | 1yr | bulk | 3mo | 6mo | 1yr | bulk | 3mo | 6mo | 1yr | bulk | 3mo | 6mo | 1yr | bulk | 3mo | 6mo | 1yr | bulk |
| **AW** | 0.530 | 0.528 | 0.562 | 0.484 | 0.544 | 0.476 | 0.505 | 0.434 | 0.492 | 0.507 | 0.539 | 0.459 | 0.527 | 0.446 | 0.477 | 0.415 | 0.459 | 0.418 | 0.442 | 0.395 | 0.430 | 0.414 | 0.440 | 0.390 | 0.426 |
| **BLO** | 0.444 | 0.543 | 0.510 | 0.495 | 0.578 | 0.472 | 0.437 | 0.426 | 0.505 | 0.542 | 0.512 | 0.498 | 0.578 | 0.505 | 0.475 | 0.463 | 0.531 | 0.451 | 0.421 | 0.415 | 0.471 | 0.456 | 0.424 | 0.410 | 0.478 |
| **CAM** | 0.057 | 0.005 | 0.015 | 0.001 | 0.034 | 0.000 | 0.000 | 0.000 | 0.000 | 0.000 | 0.000 | 0.000 | 0.000 | 0.000 | 0.005 | 0.000 | 0.005 | 0.000 | 0.000 | 0.000 | 0.000 | 0.000 | 0.000 | 0.000 | 0.007 |
| **CON** | 0.363 | 0.525 | 0.456 | 0.430 | 0.524 | 0.445 | 0.386 | 0.353 | 0.442 | 0.495 | 0.424 | 0.396 | 0.490 | 0.415 | 0.369 | 0.340 | 0.409 | 0.415 | 0.370 | 0.341 | 0.409 | 0.432 | 0.362 | 0.331 | 0.419 |
| **ET** | 0.417 | 0.457 | 0.460 | 0.551 | 0.354 | 0.180 | 0.201 | 0.288 | 0.162 | 0.323 | 0.334 | 0.466 | 0.266 | 0.274 | 0.289 | 0.382 | 0.229 | 0.281 | 0.299 | 0.404 | 0.230 | 0.319 | 0.332 | 0.444 | 0.252 |
| **TRI** | 0.562 | 0.671 | 0.634 | 0.638 | 0.644 | 0.566 | 0.545 | 0.558 | 0.567 | 0.649 | 0.605 | 0.611 | 0.620 | 0.499 | 0.497 | 0.499 | 0.500 | 0.508 | 0.508 | 0.505 | 0.507 | 0.531 | 0.524 | 0.528 | 0.527 |
| **MB** | 0.208 | 0.126 | 0.132 | 0.092 | 0.244 | 0.070 | 0.083 | 0.069 | 0.195 | 0.080 | 0.092 | 0.063 | 0.222 | 0.077 | 0.087 | 0.074 | 0.187 | 0.074 | 0.085 | 0.073 | 0.184 | 0.072 | 0.089 | 0.071 | 0.195 |
| **PG** | 0.450 | 0.306 | 0.374 | 0.299 | 0.347 | 0.138 | 0.189 | 0.110 | 0.169 | 0.158 | 0.202 | 0.141 | 0.166 | 0.165 | 0.204 | 0.166 | 0.157 | 0.169 | 0.208 | 0.175 | 0.161 | 0.181 | 0.234 | 0.187 | 0.191 |
| **SM** | 0.398 | 0.519 | 0.496 | 0.381 | 0.362 | 0.451 | 0.426 | 0.335 | 0.303 | 0.509 | 0.489 | 0.373 | 0.355 | 0.421 | 0.409 | 0.337 | 0.306 | 0.422 | 0.406 | 0.331 | 0.299 | 0.432 | 0.422 | 0.336 | 0.308 |
| **SQ** | 0.257 | 0.366 | 0.376 | 0.536 | 0.312 | 0.326 | 0.326 | 0.491 | 0.262 | 0.367 | 0.369 | 0.534 | 0.307 | 0.323 | 0.332 | 0.474 | 0.262 | 0.323 | 0.323 | 0.475 | 0.261 | 0.315 | 0.324 | 0.474 | 0.257 |

**Table S7** Values of Pearson correlation from comparison of pairwise F_ST_ values (as described by Weir and Cockerham (1984)) computed from haplotype frequencies uncovered by SSIZ or HTSA, per pipeline and sample type (ebDNA after 3 months, 6 months or 1 year of storage and bulk DNA). All correlations were significant (Mantel test; P < 0.001).

|  | bulkDNA | ebDNA 3m | ebDNA 6m | ebDNA 1y |
| --- | --- | --- | --- | --- |
| dada2 | 0.960 | 0.941 | 0.962 | 0.867 |
| obitools | 0.943 | 0.936 | 0.957 | 0.868 |
| vsearch | 0.950 | 0.929 | 0.949 | 0.852 |
| obi+swarm | 0.940 | 0.916 | 0.939 | 0.854 |
| vs+swarm | 0.953 | 0.917 | 0.941 | 0.849 |
| mothur | 0.957 | 0.933 | 0.965 | 0.872 |


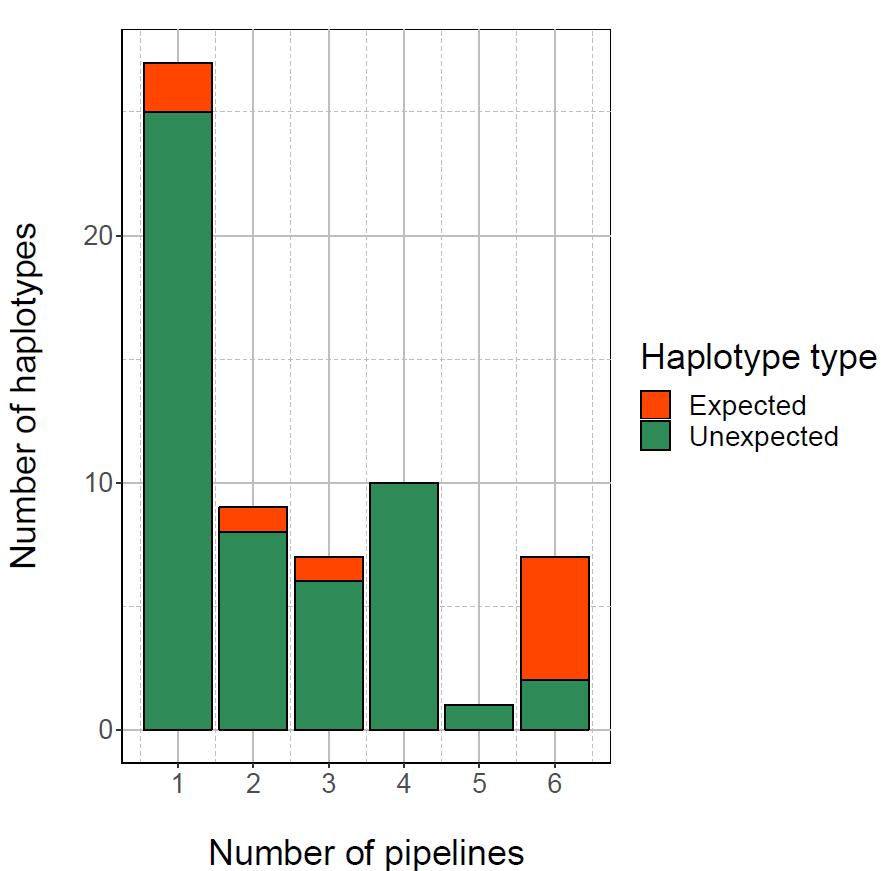


**Figure S4** Distribution of the number of expected (red) and unexpected (green) haplotypes identified by one, two, three, four, five or the six tested pipelines.


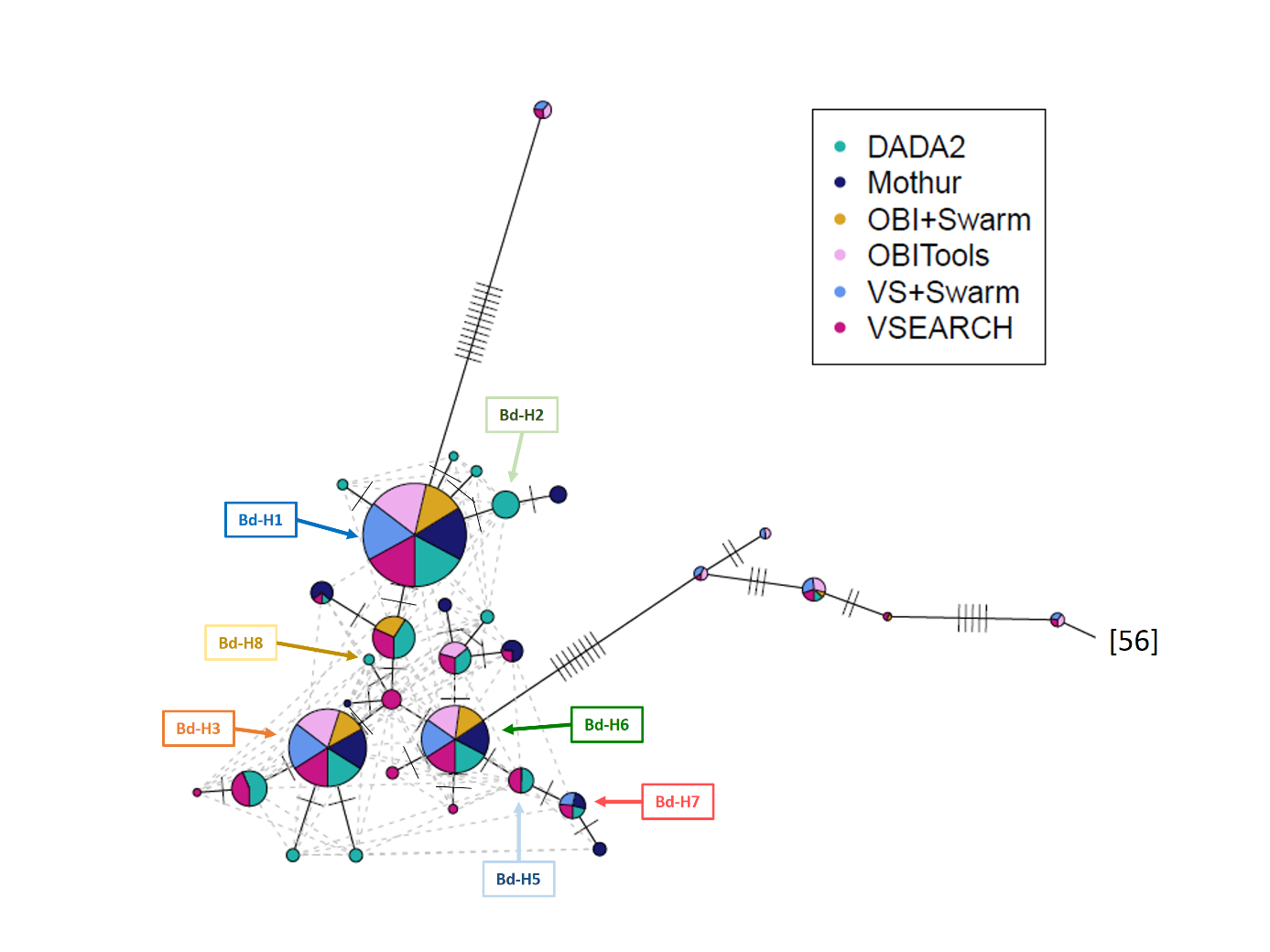


**Figure S5** Haplotype network for Botrylloides diegensis COI sequences performed with ASVs and OTUs produced by all six bioinformatics pipelines tested in this study. The size of a node represents the abundance (fourth root of the number of reads) of the corresponding ASV/OTU across all sample types. The contribution of each pipeline to the total amount of reads of each ASV/OTU is illustrated by the pie chart inside nodes. Expected haplotypes (i.e. haplotypes found with Sanger Sequencing of Individual Zooid, SSIZ) are labelled. The number of crossing lines represents the number of mutations between two nodes. The dashed grey lines figure alternative links. For a better visualization, only alternative links of one or two mutation steps are drawn. The link between B. diegensis and B. violaceus (presented in Fig. S6) has been cut for visualization purposes and the 56 mutations separating the two species are written into brackets.


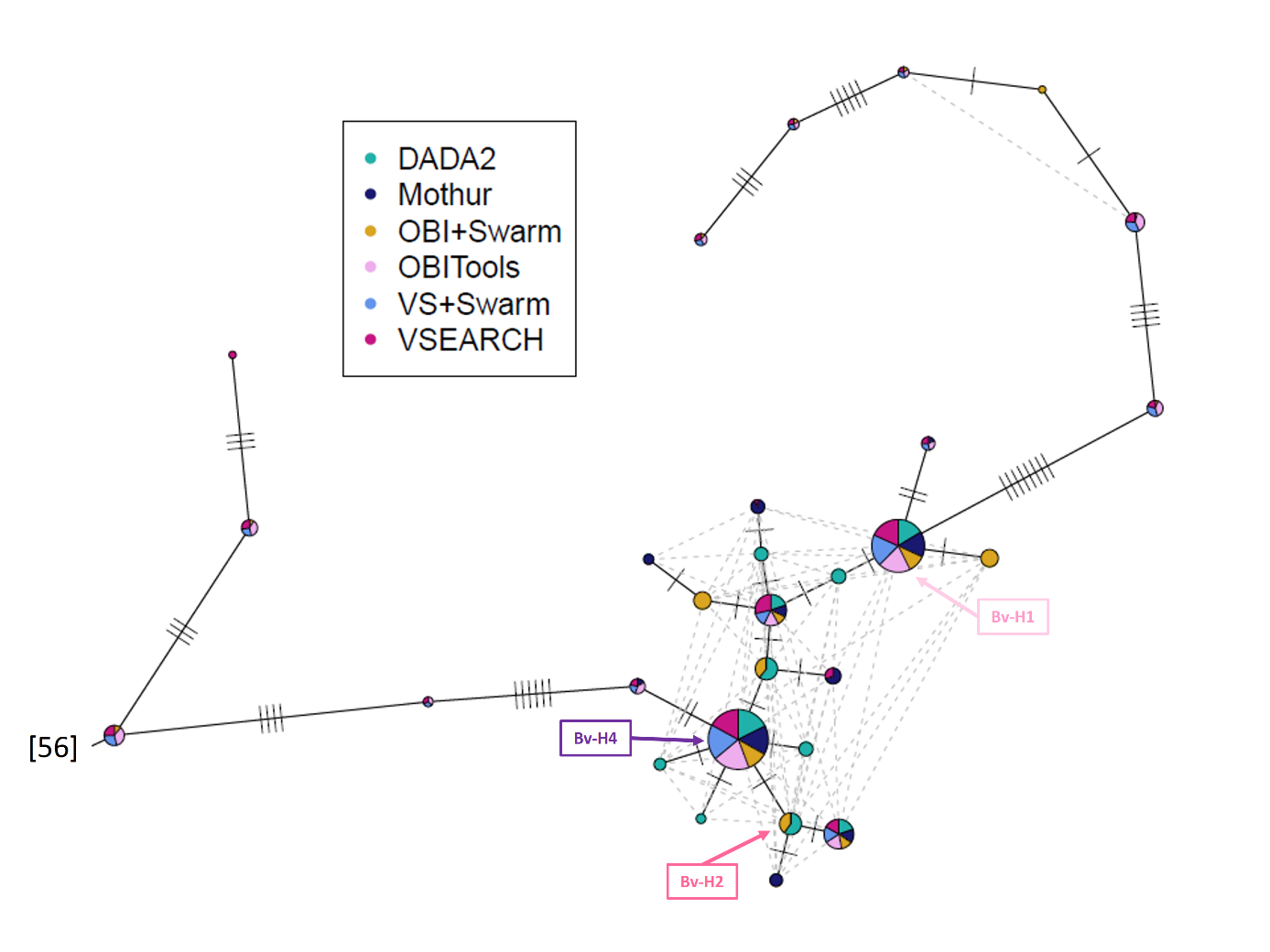


**Figure S6** Haplotype network for Botrylloides violaceus COI sequences performed with ASVs and OTUs produced by all six bioinformatics pipelines tested in this study. The size of a node represents the abundance (fourth root of the number of reads) of the corresponding ASV/OTU across all sample types. The contribution of each pipeline to the total amount of reads of each ASV/OTU is illustrated by the pie chart inside nodes. Expected haplotypes (i.e. haplotypes found with Sanger Sequencing of Individual Zooid, SSIZ) are labelled. Bv-H2 is labelled with a star because, despite being known from previous work (Viard, Roby, Turon, Bouchemousse, & Bishop, 2019), this haplotype was not found with SSIZ. The number of crossing lines represents the number of mutations between two nodes. The dashed grey lines figure alternative links. For a better visualization, only alternative links of one or two mutation steps are drawn. The link between B. violaceus and B. diegensis (presented in Fig. S5) has been cut for visualization purposes and the 56 mutations separating the two species are written into brackets.


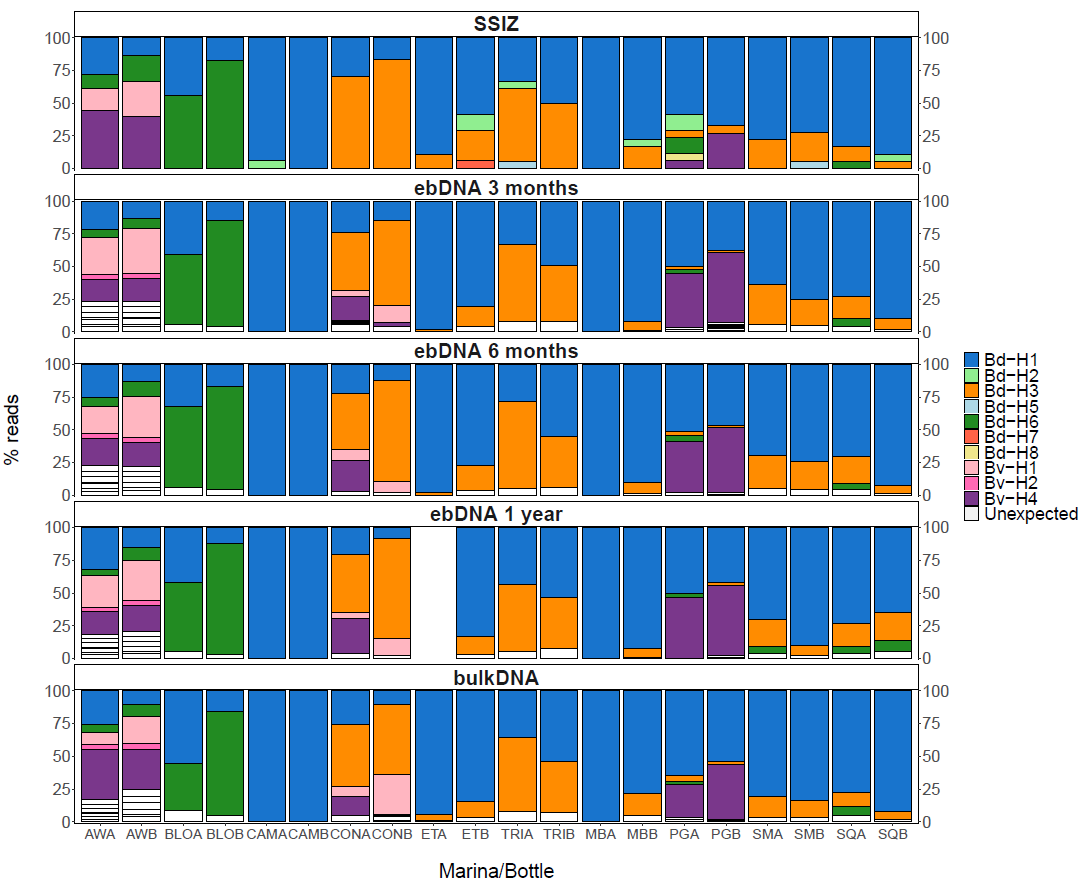


**Figure S7** Proportion of reads (or colonies in SSIZ) associated (100% identity) with each haplotype in every community (jars A and B for each location) as revealed by SSIZ (top panel) or HTSA using **obitools** for the four types of samples (four lower panels). The sample ETA after one-year storage could not be amplified. See Fig. 1 for marina codes and abbreviations of samples.


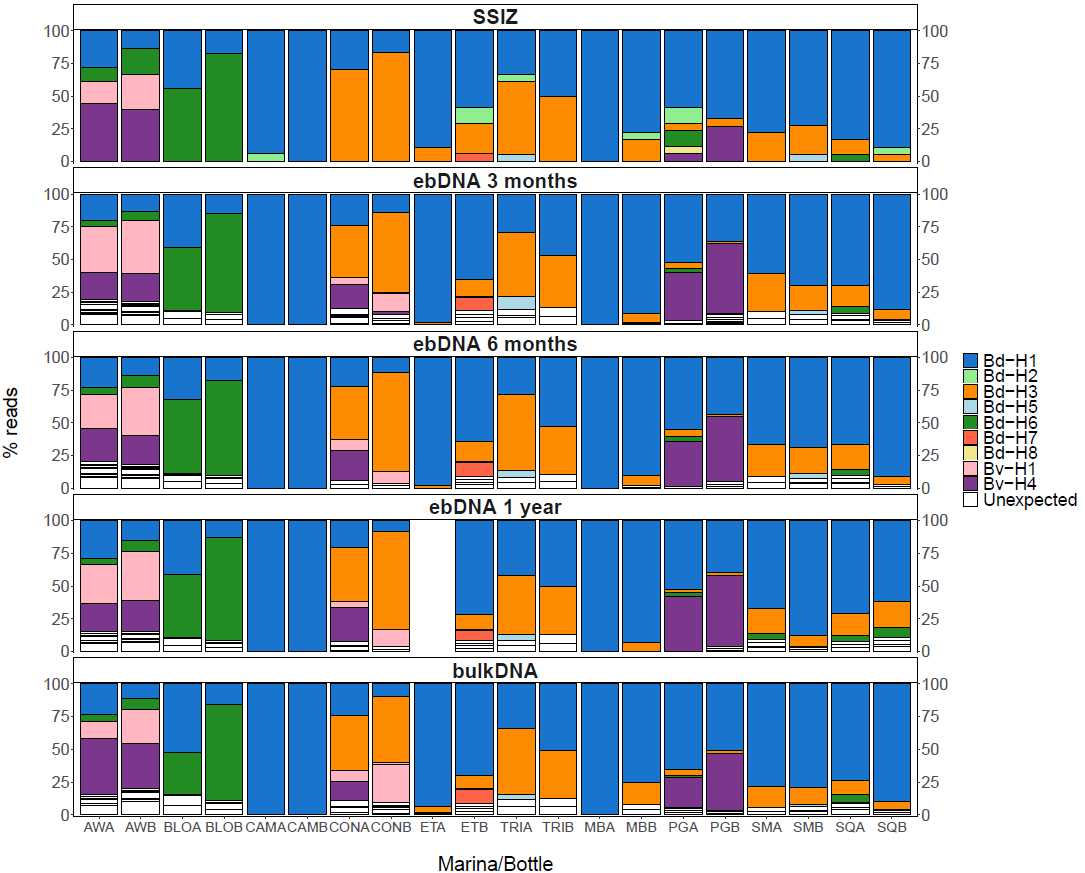


**Figure S8** Proportion of reads (or colonies in SSIZ) associated (100% identity) with each haplotype in every community (jars A and B for each location) as revealed by SSIZ (top panel) or HTSA using **vsearch** for the four types of samples (four lower panels). The sample ETA after one-year storage could not be amplified. See Fig. 1 for marina codes and abbreviations of samples.


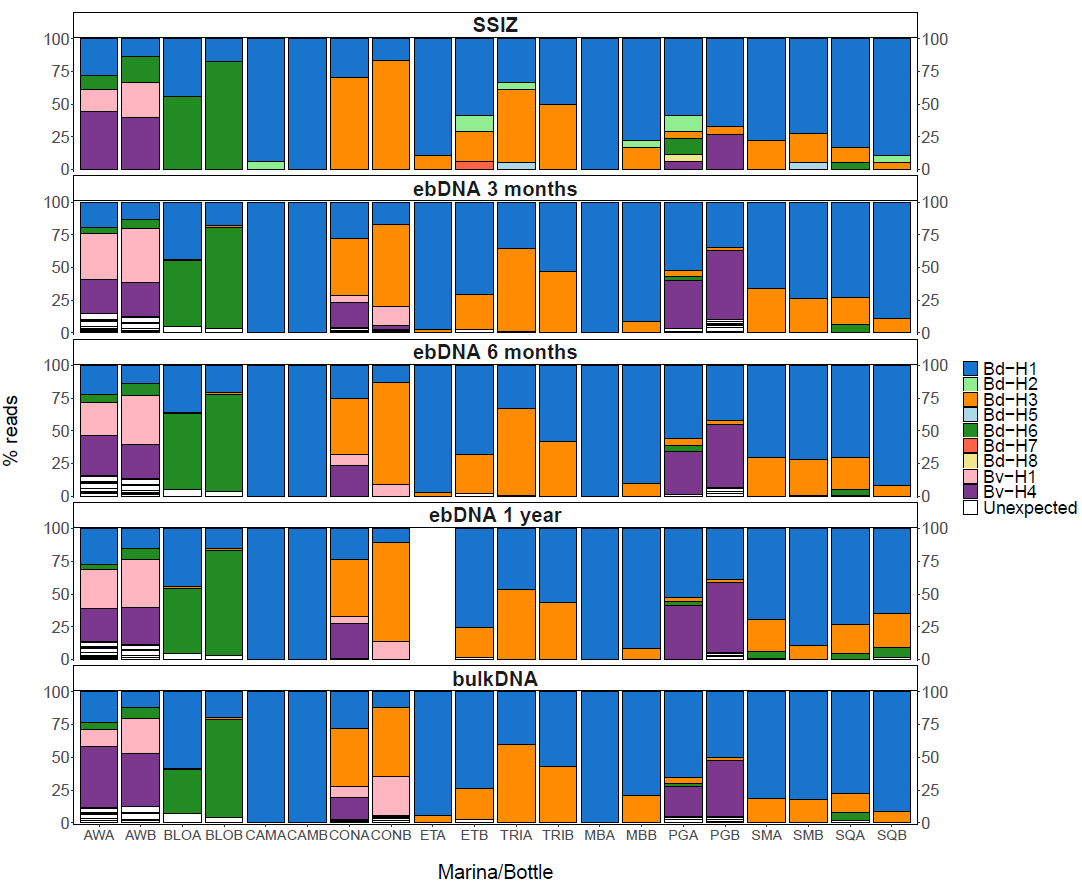


**Figure S9** Proportion of reads (or colonies in SSIZ) associated (100% identity) with each haplotype in every community (jars A and B for each location) as revealed by SSIZ (top panel) or HTSA using **obi+swarm** for the four types of samples (four lower panels). The sample ETA after one-year storage could not be amplified. See Fig. 1 for marina codes and abbreviations of samples.


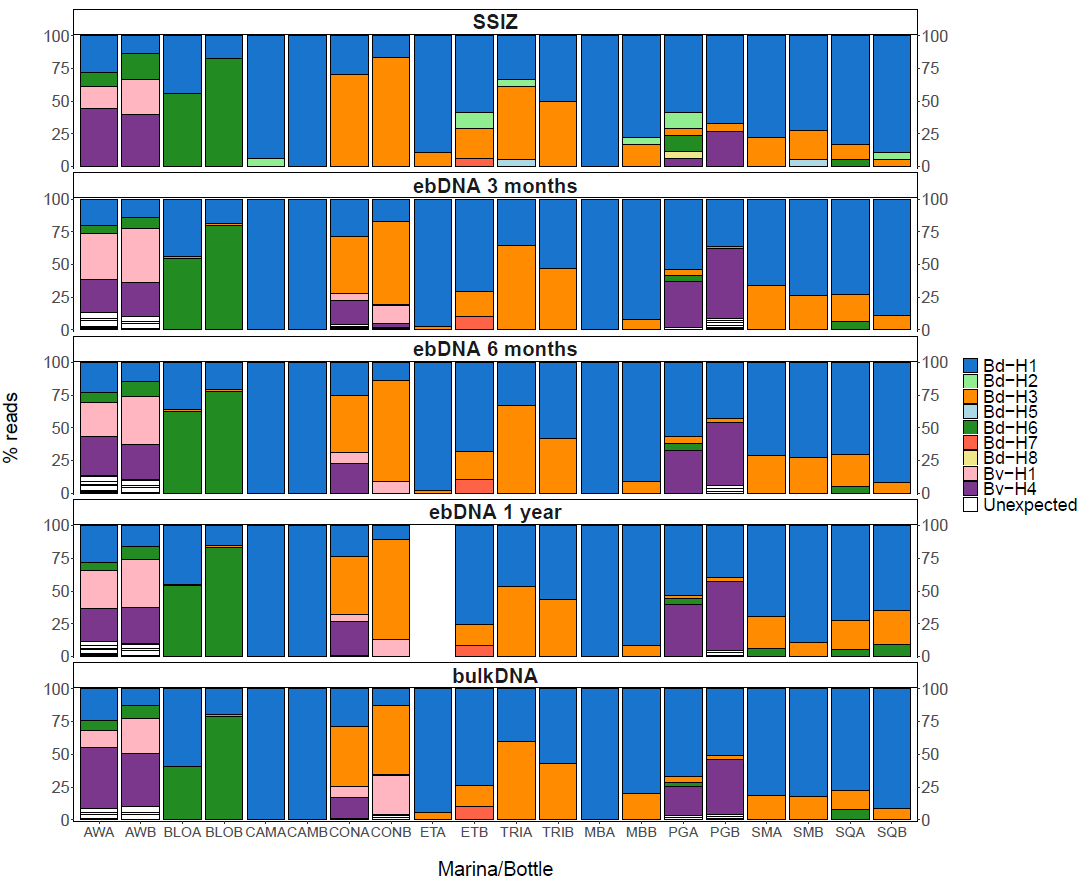


**Figure S10** Proportion of reads (or colonies in SSIZ) associated (100% identity) with each haplotype in every community (jars A and B for each location) as revealed by SSIZ (top panel) or HTSA using **vs+swarm** for the four types of samples (four lower panels). The sample ETA after one-year storage could not be amplified. See Fig. 1 for marina codes and abbreviations of samples.


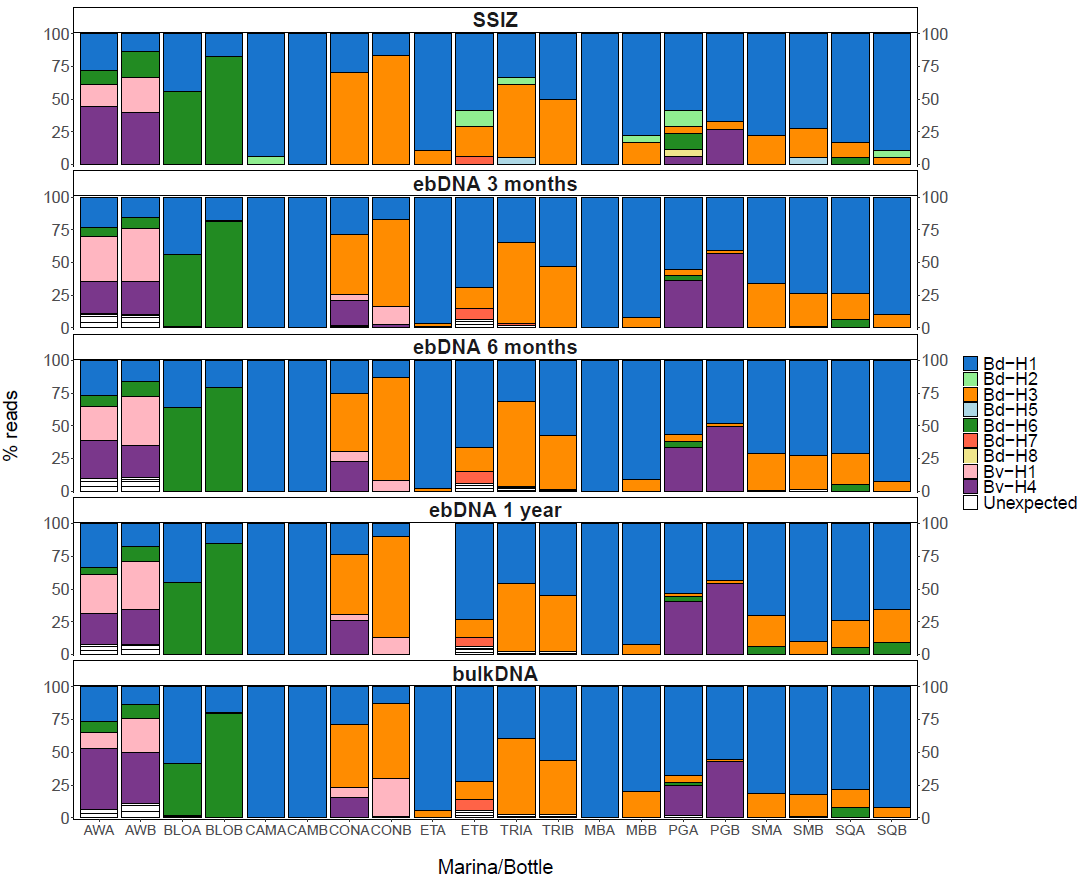


**Figure S11** Proportion of reads (or colonies in SSIZ) associated (100% identity) with each haplotype in every community (jars A and B for each location) as revealed by SSIZ (top panel) or HTSA using **mothur** for the four types of samples (four lower panels). The sample ETA after one-year storage could not be amplified. See Fig. 1 for marina codes and abbreviations of samples.


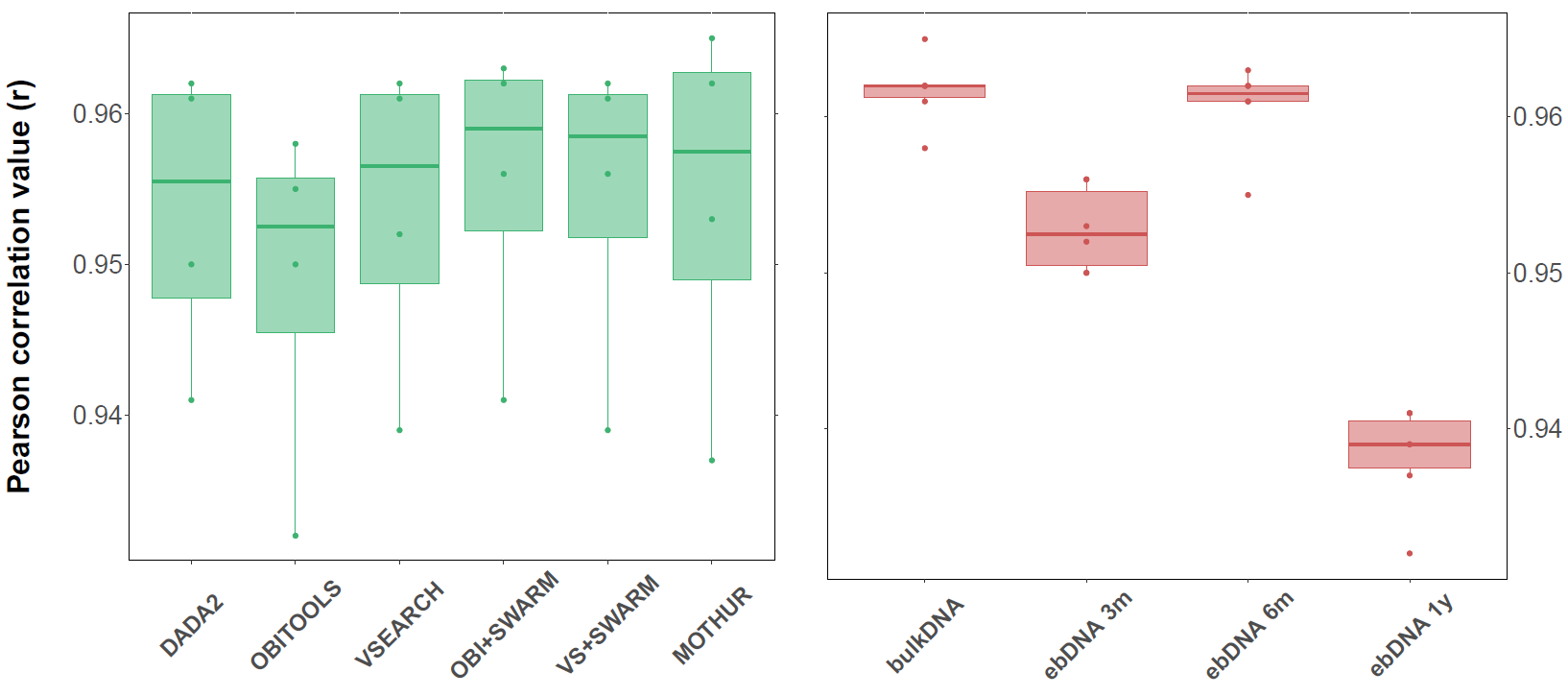


**Figure S12** Distribution of Pearson correlation coefficient r values for each pipeline (green) and each type of sample (red), computed on the relative abundance of reads of a given ASV/OTU in a given community (jar) and the proportion of colonies with this haplotype as determined by SSIZ in that community. None of the pairwise comparisons were found significant for all pairs of pipelines (Wilcoxon tests for paired samples, p-values corrected for false discovery rates ranging from 0.38 to 1.00). All pairwise comparisons were significant for all type of sample pairs (Wilcoxon tests for paired samples, p-values corrected for false discovery rates, P < 0.05), except for the comparison between bulkDNA and ebDNA 6m (P = 0.28).


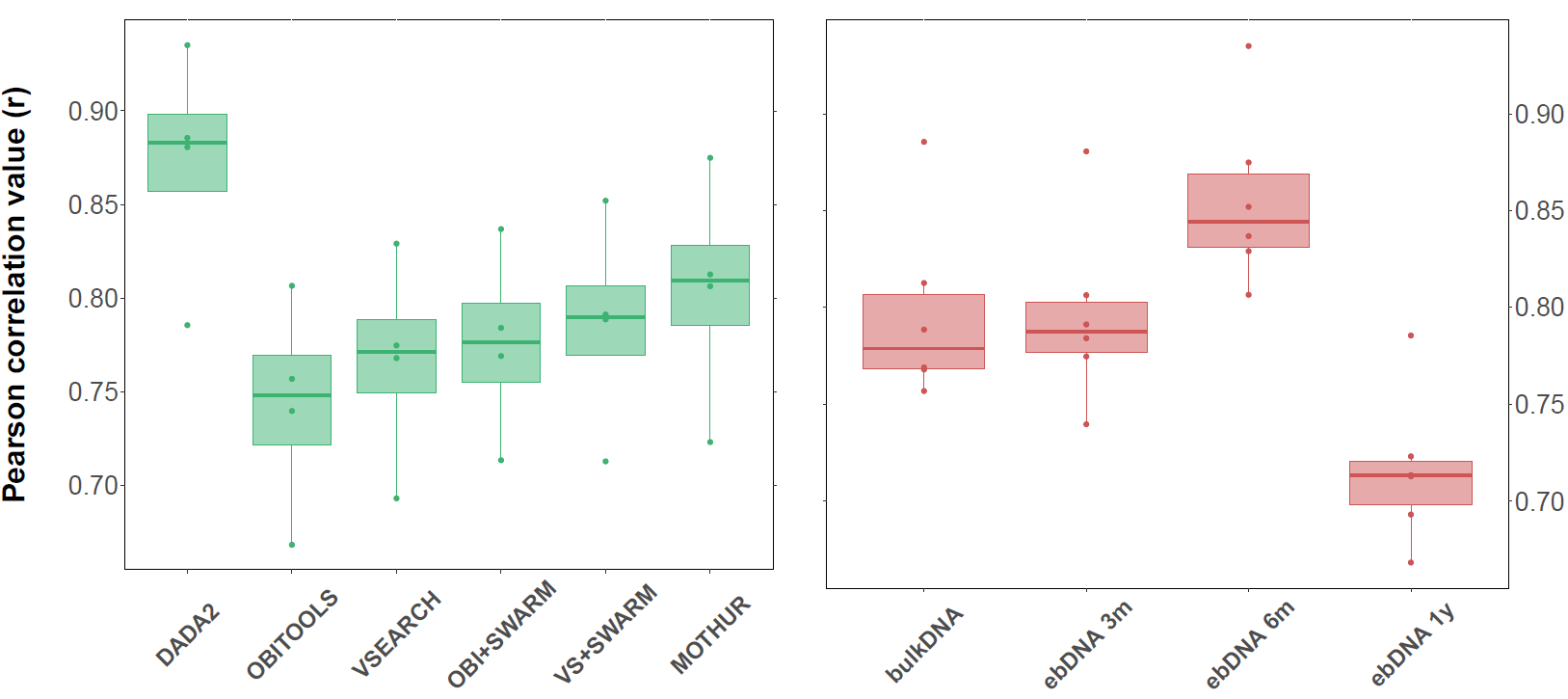


**Figure S13** Distribution of Pearson correlation coefficient r values for each pipeline (green) and each type of sample (red), between gene diversity per locus estimates (Hs) computed per locality from SSIZ and HTSA datasets. None of the pairwise comparisons were found significant different for all pairs of pipelines (Wilcoxon tests for paired samples, p-values corrected for false discovery rates ranging from 0.13 to 0.25). All pairwise comparisons were significantly different for all type of sample pairs (Wilcoxon tests, p-values corrected for false discovery rates; P < 0.05), except for the comparison between bulkDNA and ebDNA 3m (P = 1.00).

# References

Benjamini, Y. & Yekutieli, D. (2001). The control of the false discovery rate in multiple testing under dependency. The Annals of Statistics, 29(4), 1165-1188 doi: jstor.org/stable/2674075.

Boyer, F., Mercier, C., Bonin, A., Le Bras, Y., Taberlet, P. & Coissac, E. (2016). OBITOOLS: a UNIX-inspired software package for DNA metabarcoding. Molecular Ecology Resources, 16(1), 176-182 doi: 10.1111/1755-0998.12428.

Calderón-Sanou, I., Münkemüller, T., Boyer, F., Zinger, L. & Thuiller, W. (2020). From environmental DNA sequences to ecological conclusions: How strong is the influence of methodological choices? Journal of Biogeography, 47(1), 193-206 doi: 10.1111/jbi.13681.

Callahan, A. G., Deibel, D., McKenzie, C. H., Hall, J. R. & Rise, M. L. (2010). Survey of harbours in Newfoundland for indigenous and non-indigenous ascidians and an analysis of their cytochrome c oxidase I gene sequences. Aquatic Invasions, 5(1), 31-39 doi: 10.3391/ai.2010.5.1.5.

Collins, R. A., Bakker, J., Wangensteen, O. S., Soto, A. Z., Corrigan, L., Sims, D. W., ... Mariani, S. (2019). Non-specific amplification compromises environmental DNA metabarcoding with COI. Methods in Ecology and Evolution, 10(11), 1985-2001 doi: 10.1111/2041-210x.13276.

Couton, M., Comtet, T., Le Cam, S., Corre, E. & Viard, F. (2019). Metabarcoding on planktonic larval stages: an efficient approach for detecting and investigating life cycle dynamics of benthic aliens. Management of Biological Invasions, 10(4), 657-689 doi: 10.3391/mbi.2019.10.4.06

Darling, J. A., Galil, B. S., Carvalho, G. R., Rius, M., Viard, F. & Piraino, S. (2017). Recommendations for developing and applying genetic tools to assess and manage biological invasions in marine ecosystems. Marine Policy, 85, 54-64 doi: 10.1016/j.marpol.2017.08.014.

Folmer, O., Black, M., Hoeh, W., Lutz, R. & Vrijenhoek, R. (1994). DNA primers for amplification of mitochondrial cytochrome c oxidase subunit I from diverse metazoan invertebrates. Molecular marine biology and biotechnology, 3(5), 294-299 doi.

Kelly, R. P., O'Donnell, J. L., Lowell, N. C., Shelton, A. O., Samhouri, J. F., Hennessey, S. M., ... Williams, G. D. (2016). Genetic signatures of ecological diversity along an urbanization gradient. PeerJ, 4, e2444 doi: 10.7717/peerj.2444.

Leray, M., Yang, J. Y., Meyer, C. P., Mills, S. C., Agudelo, N., Ranwez, V., ... Machida, R. J. (2013). A new versatile primer set targeting a short fragment of the mitochondrial COI region for metabarcoding metazoan diversity: application for characterizing coral reef fish gut contents. Frontiers in Zoology, 10(1), 34 doi: 10.1186/1742-9994-10-34.

Nei, M. (1973). Analysis of gene diversity in subdivided populations. Proceedings of the National Academy of Sciences, 70(12), 3321-3323 doi: 10.1073/pnas.70.12.3321.

Viard, F., Roby, C., Turon, X., Bouchemousse, S. & Bishop, J. D. D. (2019). Cryptic diversity and database errors challenge non-indigenous species surveys: an illustration with *Botrylloides* spp. in the English Channel and Mediterranean Sea. Frontiers in Marine Science, 6, 615 doi: 10.3389/fmars.2019.00615.

Weir, B. S. & Cockerham, C. C. (1984). Estimating f-statistics for the analysis of population structure. Evolution, 38(6), 1358-1370 doi: 10.2307/2408641.
